# Gene expression evolution in pattern-triggered immunity within *Arabidopsis thaliana* and across Brassicaceae species

**DOI:** 10.1093/plcell/koab073

**Published:** 2021-03-05

**Authors:** Thomas M Winkelmüller, Frederickson Entila, Shajahan Anver, Anna Piasecka, Baoxing Song, Eik Dahms, Hitoshi Sakakibara, Xiangchao Gan, Karolina Kułak, Aneta Sawikowska, Paweł Krajewski, Miltos Tsiantis, Ruben Garrido-Oter, Kenji Fukushima, Paul Schulze-Lefert, Stefan Laurent, Paweł Bednarek, Kenichi Tsuda

**Affiliations:** 1 State Key Laboratory of Agricultural Microbiology, College of Plant Science and Technology, Interdisciplinary Science Research Institute, Huazhong Agricultural University, 430070 Wuhan, China; 2 The Provincial Key Lab of Plant Pathology of Hubei Province, Huazhong Agricultural University, 430070 Wuhan, China; 3 Department of Plant–Microbe Interactions, Max Planck Institute for Plant Breeding Research, 50829 Cologne, Germany; 4 Institute of Bioorganic Chemistry, Polish Academy of Sciences, 61-704 Poznan, Poland; 5 Department of Comparative Development and Genetics, Max Planck Institute for Plant Breeding Research, 50829 Cologne, Germany; 6 RIKEN Center for Sustainable Resource Science, 230-0045 Yokohama, Japan; 7 Department of Mathematical and Statistical Methods, Poznań University of Life Sciences, 60-628 Poznań, Poland; 8 Institute of Bioorganic Chemistry, Polish Academy of Sciences, 61-704 Poznań, Poland; 9 Institute of Plant Genetics, Polish Academy of Sciences, 60-479 Poznań, Poland; 10 Institute for Molecular Plant Physiology and Biophysics, University of Würzburg, 97082 Würzburg, Germany; 11 Graduate School of Bioagricultural Sciences, Nagoya University, Nagoya 464-8601, Japan

## Abstract

Plants recognize surrounding microbes by sensing microbe-associated molecular patterns (MAMPs) to activate pattern-triggered immunity (PTI). Despite their significance for microbial control, the evolution of PTI responses remains largely uncharacterized. Here, by employing comparative transcriptomics of six *Arabidopsis thaliana* accessions and three additional Brassicaceae species to investigate PTI responses, we identified a set of genes that commonly respond to the MAMP flg22 and genes that exhibit species-specific expression signatures. Variation in flg22-triggered transcriptome responses across Brassicaceae species was incongruent with their phylogeny, while expression changes were strongly conserved within *A. thaliana*. We found the enrichment of WRKY transcription factor binding sites in the 5′-regulatory regions of conserved and species-specific responsive genes, linking the emergence of WRKY-binding sites with the evolution of gene expression patterns during PTI. Our findings advance our understanding of the evolution of the transcriptome during biotic stress.

## Teaser

Closely related Brassicaceae plants show considerable variation in gene expression patterns during pattern-triggered immunity.

##  

**Figure:**
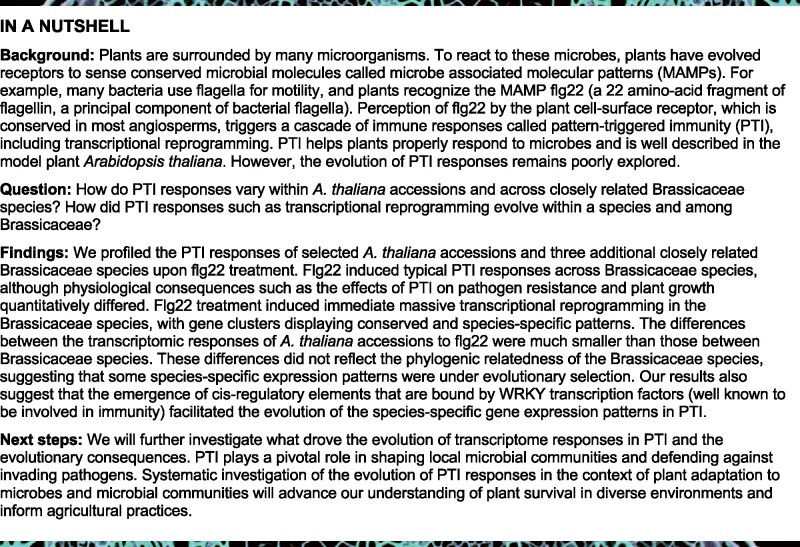


## Introduction

The evolution of biological traits is determined both by variation in coding sequence and gene expression (Necsulea and Kaessmann, 2014; Das Gupta and Tsiantis, 2018). However, our understanding of gene expression variation remains fragmented. The conservation of gene expression patterns over millions of years across species points to the general importance of such expression patterns and indicates an evolutionary constraint (Cardoso-Moreira et al., 2019). Conversely, diversified gene expression patterns across different species may reflect neutral or adaptive evolution (Harrison et al., 2012). The immanent noise in expression data makes it difficult to disentangle environmental and genetic effects on gene expression variation; therefore, detecting gene expression variation caused by genetic effects requires comparisons to be performed under the same experimental conditions (Voelckel et al., 2017).

In previous comparative transcriptome studies in plants, it was noted that variation in gene expression between or within species was substantially enriched for stress-responsive genes, which suggests that changes in stress-responsive gene expression may play an important role in adaptation to the environment (Koenig et al., 2013; Voelckel et al., 2017; Groen et al., 2020). Despite this notion, little is known about how stress-induced transcriptomic changes evolved in plants. To date, few studies comparing expression variation within and across species in unified experimental setups have been performed, especially for plants.

In recent years, several close and distant relatives of the model plant *Arabidopsis thaliana*, belonging to the Brassicaceae family, have been subjected to genome sequencing and used as model systems to study the evolution of various biological traits. For instance, a comparison of the hairy bittercress (*Cardamine hirsuta*) genome with that of *A. thaliana* increased our understanding of the molecular mechanisms that mediate the evolution of leaf shapes and pod shattering (Vlad et al., 2014; Gan et al., 2016; Das Gupta and Tsiantis, 2018). The genome sequences of other Brassicaceae species including pink shepherd’s-purse (*Capsella rubella*) and saltwater cress (*Eutrema salsugineum*) have been used to analyze the mechanisms underlying selfing and abiotic stress-tolerance, respectively (Wu et al., 2012; Slotte et al., 2013; Yang et al., 2013). The availability of rich genomic resources, the broad phylogenetic representations, and the feasibility of growing these Brassicaceae species under the same experimental conditions make them excellent systems for comparative genomics, transcriptomics, and metabolomics analyses.

In nature, plants are surrounded by microbes that could potentially be beneficial or pathogenic (Fitzpatrick et al., 2020). To properly respond to the presence of these microbes, plants have evolved cell-surface localized pattern recognition receptors (PRRs) that sense conserved microbe-associated molecular patterns (MAMPs), leading to the activation of pattern-triggered immunity (PTI) (Albert et al., 2020; Zhou and Zhang, 2020). The two best-characterized MAMPs are the bacteria-derived oligopeptides flg22 and elf18, which are sensed by their corresponding leucine-rich repeat PRRs FLAGELLIN SENSING 2 (FLS2) and EF-TU RECEPTOR (EFR), respectively, in *A. thaliana*. Treating plants with flg22 or elf18 elicits a set of temporally coordinated responses including rapid MAP kinase (MAPK) phosphorylation, genome-wide transcriptional reprogramming, and phytohormone and secondary metabolite production, followed by inhibition of plant growth and increased resistance against pathogens (Albert et al., 2020). Although PTI has been extensively scrutinized in the context of plant–pathogen interactions, it was also recently implicated in the assembly of the plant microbiota, a set of microbes with taxonomically defined structure and composition that colonize the healthy plant (Hacquard et al., 2017; Chen et al., 2020). Thus, PTI serves as the key mechanism that allows plants to adapt to different environments characterized by different microbial communities.

Despite the significance of PTI for plant adaptation to the environment, our understanding of PTI evolution is limited to the evolution of PRRs. For instance, genomes of many plant lineages including members of Brassicaceae, Solanaceae, and Poaceae contain *FLS2*, whereas *EFR* appears to be restricted to Brassicaceae (Boutrot and Zipfel, 2017). However, the conservation of PTI responses among different species and how PTI responses evolve remains poorly understood. Here, we took a comparative transcriptomic and metabolomic approach using *A. thaliana* (six accessions), *C. rubella*, *C. hirsuta*, and *E. salsugineum* in a unified experimental setup with multiple time points to address the evolution of flg22-triggered responses in plants.

## Results

### The tested Brassicaceae plants respond to the MAMP flg22

**Figure 1:**
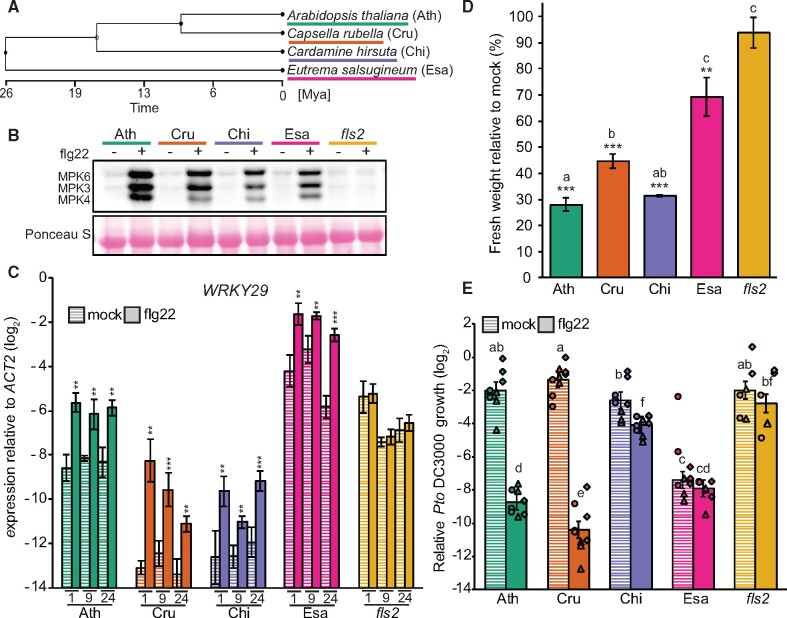
All tested Brassicaceae species sense flg22. A, Phylogenetic tree generated with TimeTree (www.timetree.org) indicating the evolutionary distances between the four Brassicaceae species used in this study. Ath, *A. thaliana* (Col-0); Cru, *C. rubella* (N22697); Chi, *C. hirsuta* (Oxford); Esa, *E. salsugineum* (Shandong). B, 12-day-old seedlings were treated with mock or 1 µM flg22 for 15 min, and MAPK phosphorylation was measured by immunoblotting using an antiP42/44 antibody. Ponceau staining is shown as a loading control. Experiments were repeated at least three times with similar results. C, Expression of *WRKY29* was determined by RT-qPCR at 1, 9, and 24 h after mock or 1 µM flg22 treatment of 12-day-old seedlings. Bars represent means and SEs of log_2_ expression levels relative to *ACTIN2* calculated from three independent experiments. Asterisks indicate significant difference from mock (mixed linear model followed by Student’s *t* test, ^**^P < 0.01; ^***^P  < 0.001). D, Seven-day-old seedlings were transferred into liquid medium containing mock or 1 µM flg22 for 12 days. The fresh weight of 12 pooled seedlings was measured. The bars represent means and SEs from three independent experiments. Relative fresh weight (%) of flg22-treated seedlings compared to mock seedlings is shown. Statistical analysis was performed with log_2_-transformed raw fresh weight values. Asterisks indicate significant flg22 effects in each genotype (mixed linear model followed by Student’s *t* test, ^**^P < 0.01; ^***^P < 0.001). Different letters indicate significant differences in flg22 effects between different genotypes (mixed linear model, adjusted *P* < 0.01). E, Five-week-old plants were syringe-infiltrated with 1 μM flg22 or mock 24 h prior to infiltration with *Pto* DC3000 (OD_600_ = 0.0002). The bacterial titer was determined 48 h after bacterial infiltration. The log_2_ ratio of copy numbers of a bacterial gene (*oprF*) and a plant gene (*ACTIN2*) was determined by qPCR and used to represent relative *Pto* DC3000 growth. Bars represent means and SEs from three independent experiments, each with three biological replicates from different inoculated plants (*n* = 9). The biological replicates from three independent experiments are represented by dots, triangles, and squares. Different letters indicate statistically significant differences (mixed linear model, adjusted *P* < 0.01).

Based on our analysis using TIMETREE (see the “Methods” section), the Brassicaceae species *C. rubella*, *C. hirsuta*, and *E. salsugineum* diverged from *A. thaliana* ∼9, 17, and 26 million years ago (Mya), respectively (Figure 1, A). We first investigated whether flg22 treatment induces rapid phosphorylation of MPK3 and MPK6, a typical early event in PTI, in these four Brassicaceae plants. Although a previous report indicates that protein extracts from *C. hirsuta*, including those from the Oxford accession (Gan et al., 2016), do not bind to flg22 (implying that *C. hirsuta* does not sense flg22) (Vetter et al., 2012), we observed a clear phosphorylation of the MAPKs MPK3 and MPK6 upon flg22 treatment in all tested Brassicaceae plants, including the Oxford accession of *C. hirsuta*, which was absent in the *A. thaliana fls2* mutant (Figure 1, B). We also observed induction of the transcription factor (TF) gene *WRKY29*, a widely used immune marker in *A. thaliana* (Asai et al., 2002), in all tested species at 1, 9, and 24 h after flg22 application (Figure 1, C). Thus, all four tested Brassicaceae species sense flg22 to trigger typical early PTI responses, as observed in *A. thaliana*.

PTI activation reduces plant growth, a late PTI response detectable days after MAMP perception, which is another common measure of PTI outputs in *A. thaliana* (Gómez-Gómez et al., 1999). With the exception of the *fls2* mutant, chronic flg22 exposure reduced seedling growth in all tested species, but the extent of flg22-triggered growth reduction varied and was significantly weaker in *E. salsugineum* compared with the other three species (Figure 1, D).

Another PTI output is enhanced pathogen resistance induced by pre-treating plants with a MAMP. For example, flg22 pre-treatment reduces proliferation of the foliar bacterial pathogen *Pseudomonas syringae* pv. *tomato* DC3000 (*Pto* DC3000) in *A. thaliana* leaves (Zipfel et al., 2004; Tsuda et al., 2008). We found that flg22 pre-treatment reduced bacterial proliferation in *A. thaliana* and *C. rubella* (Figure 1, E). In contrast, *Pto* DC3000 growth was only slightly reduced in *C. hirsuta* and was not altered in *E. salsugineum* by flg22 treatment (Figure 1, E). Thus, the robust induction of early PTI responses by flg22 observed in all tested Brassicaceae does not necessarily lead to heightened immunity against this bacterial pathogen (Figure 1, B and C). We noticed that the *Pto* DC3000 titer was much lower in *E. salsugineum* compared with the other species (Figure 1, E) and speculated that type III effector(s) from *Pto* DC3000 may be recognized in *E. salsugineum*, triggering ETI and masking the flg22-triggered PTI effect. However, flg22 pre-treatment followed by inoculation with a *Pto* DC3000 mutant strain lacking the functional type III secretion system (*Pto hrcC*) led to reduced bacterial growth in *A. thaliana* but not in *E. salsugineum* (Supplemental Figure S1). Thus, flg22 pre-treatment was ineffective against this bacterial pathogen in *E. salsugineum*. In summary, while flg22 triggers typical PTI responses in all tested Brassicaceae plants, the physiological consequences, such as plant growth inhibition and bacterial resistance, vary across species.

### Flg22 triggers extensive transcriptional reprogramming in all tested Brassicaceae species

**Figure 2:**
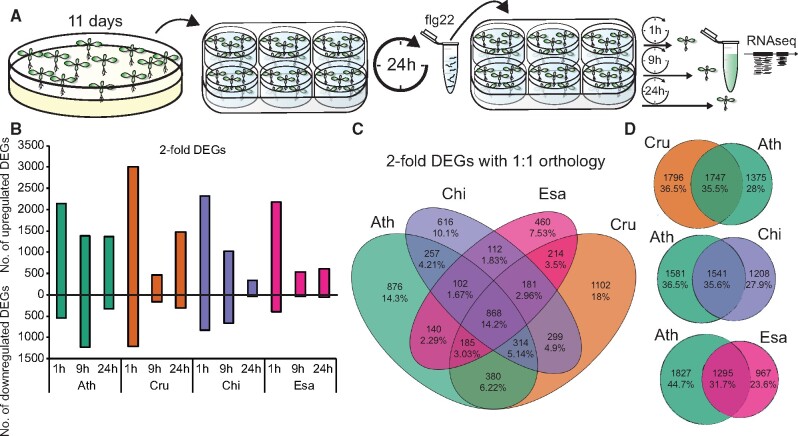
All tested Brassicaceae species trigger massive transcriptional reprogramming upon flg22 perception. A, Schematic representation of the experimental design. B, The number of DEGs (*q*-value <0.01 and |log_2_ fold change| >1) for up- or down-regulated genes was plotted at the indicated time points for each species. C, A Venn diagram showing the number of shared and specific DEGs between species. All DEGs that were differentially expressed at least at one time point in one species that showed 1:1 orthology were used. D, Venn diagrams showing the number of shared DEGs between *A. thaliana* and the indicated species. Ath, *A. thaliana* (Col-0); Cru, *C. rubella* (N22697); Chi, *C. hirsuta* (Oxford); Esa, *E. salsugineum* (Shandong).

To study the evolution of transcriptomic changes during PTI responses, we generated RNA-sequencing (RNA-seq) data for early (1 h), intermediate (9 h), and late (24 h) transcriptome responses after flg22 or mock treatment of the four Brassicaceae species (Figure 2, A). In total, this dataset comprised 72 samples with 33.3 million 100-bp strand-specific reads per sample on average. The RNA-seq reads were mapped to their corresponding genomes, and normalized and log_2_-transformed count data were used for statistical analysis using a linear model (see the “Methods” section).

We identified differentially expressed genes (DEGs) upon flg22 treatment compared with the mock samples based on an adjusted *P*-value ˂0.01 and a minimum fold-change of two for each species at each time point. We observed massive transcriptional reprogramming in all species, with 4,349, 4,964, 4,038, and 2,861 DEGs in *A. thaliana* (Ath), *C. rubella* (Cru), *C. hirsuta* (Chi), and *E. salsugineum* (Esa), respectively (Figure 2, B). The number of upregulated genes at 1 h was comparable among species, while the number of downregulated genes at 1 h was more variable, with approximately three times as many downregulated genes in *C. rubella* as in *E. salsugineum*. Interestingly, the number of DEGs at later time-points differed markedly among these species: the expression of ∼2,000 genes was altered in *A. thaliana* and *C. rubella*, whereas only 300–500 genes were differentially regulated in *C. hirsuta* and *E. salsugineum* at 24 h after flg22 treatment (Figure 2, B).

To compare the expression changes of individual genes among species, we used Best Reciprocal BLAST to detect 1:1 orthologs between *A. thaliana* and the other Brassicaceae species. Subsequently, we only selected genes showing a 1:1 orthologous relationship between *A. thaliana* and each of the Brassicaceae species, resulting in a set of 17,856 orthologous genes (Supplemental Data Set S1). Of the 6,106 genes that were differentially expressed during at least one time point in at least one of the species, 868 DEGs (14.2%) were shared among all Brassicaceae species (Figure 2, C andSupplemental Figure S2, A–C). These 868 DEGs represent a core set of flg22-responsive genes in these Brassicaceae species, as their responses to flg22 were maintained over 26 million years of evolution. We also found that a substantial number of DEGs were species-specific (Figure 2, C). The specific up- or down-regulation of 460–1,102 DEGs suggests substantial diversification of flg22-triggered transcriptional responses during Brassicaceae evolution. Comparisons between *A. thaliana* and each of the species revealed that approximately one-third of flg22-induced transcriptional changes (35.5% with *C. rubella*, 35.6% with *C. hirsuta*, and 31.7% with *E. salsugineum*) were shared between *A. thaliana* and each of the respective species (Figure 2, D). Taken together, flg22 triggers overlapping but distinct, massive transcriptional reprogramming in these Brassicaceae species.

The interpretation of comparative transcriptomics data in its current form is limited to orthologous genes. However, a portion of the transcriptomes that do not show 1:1 orthology may include genes that had uniquely emerged in certain species due to gene duplication events and functional innovations that could potentially contribute to or even drive modifications in the transcriptome landscape. To address this notion, we compared the fraction of DEGs and the magnitude of expression changes between 1:1 orthologs and non 1:1 orthologs. We found that the fraction of DEGs between the two groups was similar. A slightly higher proportion of genes that were responsive to flg22 treatment at 1 h were 1:1 orthologs, and there were no profound differences among these Brassicaceae species (Supplemental Figure S2, D). Also, the magnitude of expression changes between 1:1 orthologs and non 1:1 orthologs was similar (Supplemental Figure S2, E). Therefore, we focused on 1:1 orthologs for further analysis, as our major aim was to compare gene expression variation among these Brassicaceae species.

### Conserved flg22-responsive genes during Brassicaceae evolution

**Figure 3:**
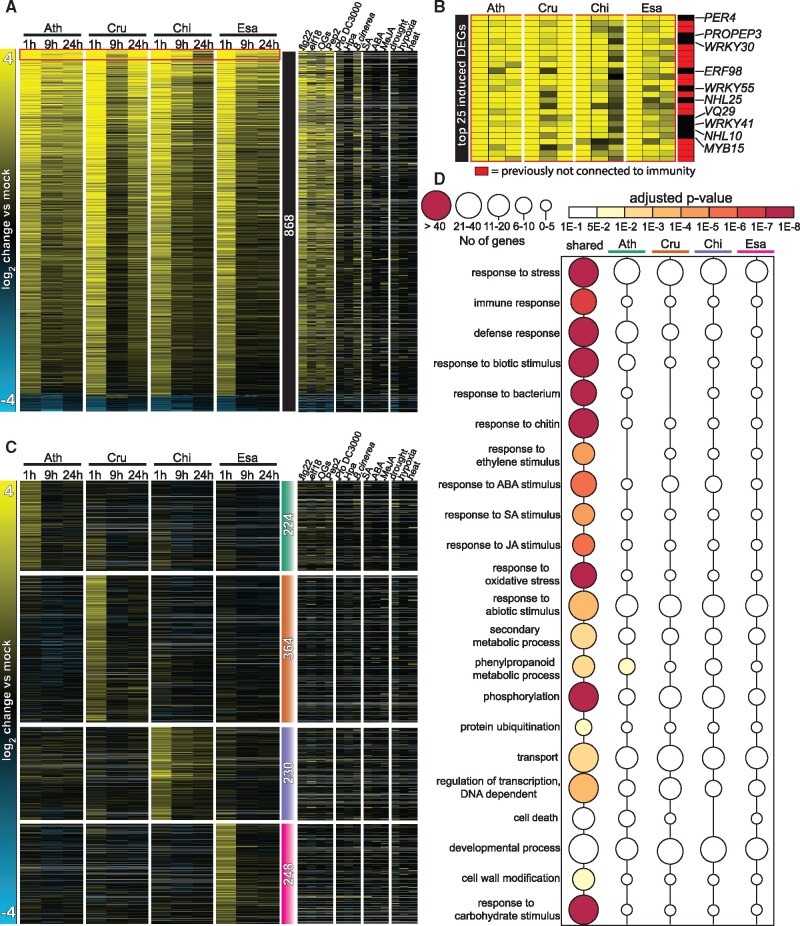
Conserved yet distinct transcriptomic responses to flg22 in Brassicaceae species. A, Heatmap of 868 DEGs shared among the four tested Brassicaceae species (see Figure 2, C) sorted by mean expression values. The heatmap on the right displays expression changes of the 868 DEGs under the indicated stress conditions in publicly available *A. thaliana* datasets (Genevestigator). Ath, *A. thaliana* (Col-0); Cru, *C. rubella* (N22697); Chi, *C. hirsuta* (Oxford); Esa, *E. salsugineum* (Shandong). See Supplemental Data Set S2 for a list of individual genes. B, Heatmap of top 25 flg22-induced genes based on the mean induction of all samples. Red indicates DEGs that previously have not been implicated in plant immunity. C, All 6,106 DEGs were clustered by *k*-means (*k* = 15), and four clusters exhibiting species-specific expression signatures are shown (see Supplemental Data Set S3). Colored bars with the number of genes indicate Ath- (green), Cru- (orange), Chi- (purple), and Esa- (magenta) specific clusters. The heatmap on the right displays expression changes of these genes under the indicated stress conditions in publicly available *A. thaliana* datasets (Genevestigator). See Supplemental Data Set S3 for a list of individual genes. D, Enrichment of selected GO terms among common DEGs and species-specific expression clusters (generated with BinGO). Circle sizes indicate the number of genes within each GO term and the color of the circle indicates the adjusted *P*-values for the enrichment of the respective GO terms. See Supplemental Data Set S4 for the full GO terms.

Next, we examined the expression dynamics of the 868 shared DEGs (Figure 3, A andSupplemental Data Set S2). These shared DEGs exhibit similar expression patterns among all four species: genes induced in one species were also induced in the other species. Comparisons with publicly available datasets revealed that these shared genes are commonly responsive to MAMPs (flg22 and elf18) and damage-associated molecular patterns (oligogalacturonides [OGs] and Pep2) in *A. thaliana* (Figure 3, A). Many well-known genes involved in different aspects of plant immunity were among the conserved flg22-responsive genes, such as genes involved in MAMP perception (*CERK1*, *BAK1*, *BIK1*, and *SOBIR1*), reactive oxygen species production (*RBOHD*), MAPK cascades (*MKK4* and *MPK3*), salicylic acid (SA) signaling (*CBP60G*, *NPR1*, and *NPR3*), and immune-related TFs (*WRKY13/33/40/62*, *ERF6/104*, and *MYB51/122*) (Figure 3, B). In addition, a large number of genes, i.e. ∼50% of the top 25 induced genes, whose high induction by flg22 was conserved among all tested Brassicaceae, were either functionally unannotated or were not previously associated with immunity (Figure 3, B; red boxes). Thus, many genes with potentially important and conserved functions in plant immunity remain to be characterized. Taken together, our data define a core set of genes with conserved flg22 responsiveness over 26 million years of Brassicaceae evolution, suggesting that the regulation of these genes might be broadly relevant for plant–bacterial interactions.

### Differences in flg22-triggered transcriptomic responses among Brassicaceae species

While in general, a similar number of genes were differentially expressed after flg22 treatment in the tested Brassicaceae plants, there were substantial differences in temporal dynamics. For instance, transcriptional reprogramming was more transient in *E. salsugineum* compared with *A. thaliana*, and *C. rubella* showed a peculiar pattern characterized by a decrease in the number of DEGs at 9 h and an increase at 24 h (Supplemental Figure S3, A). Rapid and sustained transcriptional responses were previously associated with effective bacterial resistance (Lu et al., 2009; Tsuda et al., 2013; Mine et al., 2018). Thus, the lack of flg22-triggered growth restriction of *Pto* DC3000 in *E. salsugineum* (Figure 1, E andSupplemental Figure S1) might be explained by the transient nature of the transcriptional response in this species. To gain insights into the biological processes associated with this expression pattern, we extracted genes that were induced at 1 h in both *A. thaliana* and *E. salsugineum* and were induced at 24 h in *A. thaliana* but not in *E. salsugineum* (Supplemental Figure S3, B). By investigating publicly available gene expression datasets, we found that most of these genes were induced by SA in *A. thaliana* (Supplemental Figure S3, C). Consistent with this analysis, flg22 treatment increased SA levels in *A. thaliana* but not in *E. salsugineum* (Supplemental Figure S3, D). These results suggest that activated SA signaling is responsible for sustained transcriptional reprogramming in *A. thaliana*. However, flg22-induced transcriptome responses were comparable between wild-type *A. thaliana* Col-0 and a mutant in *SID2*, an SA biosynthesis gene responsible for increased SA accumulation in response to flg22 (Supplemental Figure S3, E–H; Hillmer et al., 2017). Thus, SA accumulation alone does not explain the distinct temporal dynamics of transcriptional reprogramming in these Brassicaceae plants.

A considerable number of genes were only differentially expressed in one of the Brassicaceae species (Figure 2, C). To understand the degree of specificity in gene expression patterns in these Brassicaceae plants, we clustered and visualized the expression changes of all 6,106 DEGs (Supplemental Figure S4). This analysis revealed that although most of the DEGs showed similar expression patterns, four gene clusters exhibited species-specific signatures (Figure 3, C, Supplemental Figure S4, and Supplemental Data Set S3). These four clusters contained 1,086 genes, representing ∼18% of all DEGs. Although gene ontology (GO) term analysis of the shared DEGs revealed a strong enrichment of defense-associated biological processes including immune/defense response, response to bacterium, and response to ethylene/SA/JA stimulus, there was almost a complete lack of GO term enrichment within the four gene clusters showing species-specific expression signatures (Figure 3, D andSupplemental Data Set S4). Perhaps genes showing species-specific patterns may be involved in a collection of biological processes, or perhaps GO analysis is inherently biased toward functional validation and association in *A. thaliana*.

### Transcriptomic responses to flg22 are highly conserved among genetically and geographically diverse *A. thaliana* accessions

**Figure 4:**
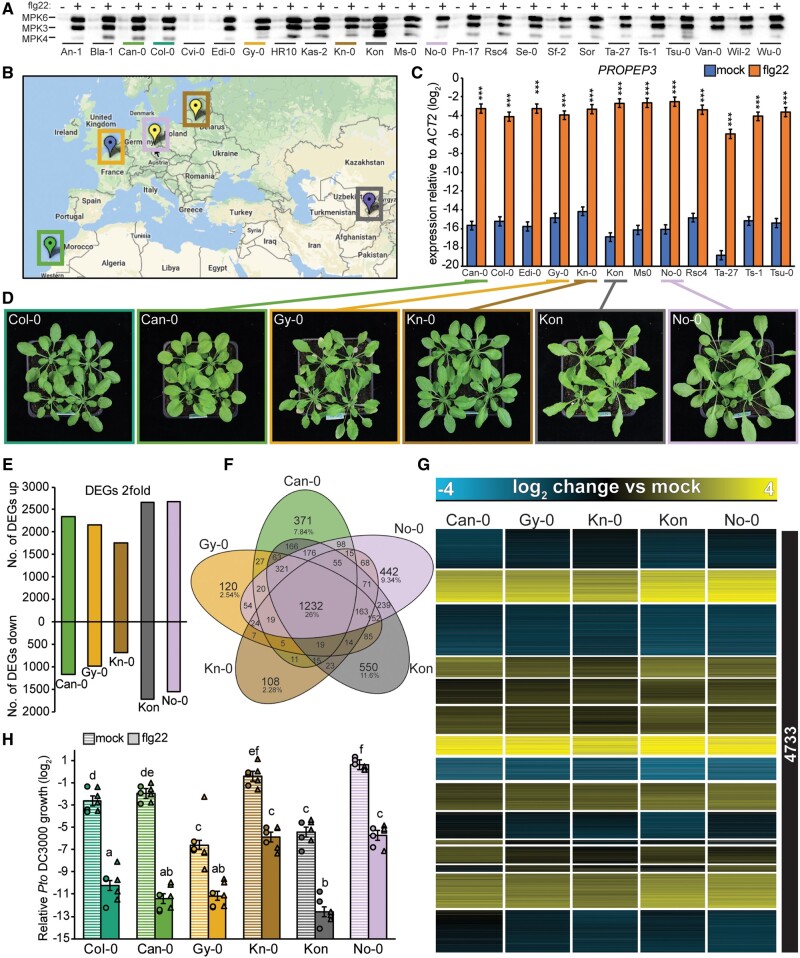
Flg22-triggered transcriptional responses show a high degree of conservation among *A. thaliana* accessions with diverse genetic backgrounds. A, Twelve-day-old seedlings were treated with mock or 1 µM flg22 for 15 min, and MAPK phosphorylation was measured in the indicated *A. thaliana* accessions by immunoblotting using an anti-p42/44 antibody. B, Geographic origins of the five accessions chosen for RNA-seq analysis are shown on the map created at 1001genomes.org. The colors of the markers indicate different genetic groups determined by The 1001 Genomes Consortium (1001 Genomes Consortium, 2016). C, Twelve-day-old *A. thaliana* seedlings were treated with mock or 1 μM flg22 for 1 h, and expression of *PROPEP3* was determined by RT-qPCR. The accessions highlighted in color were used for the RNA-seq experiments. Bars represent means and SEs of log_2_ expression levels relative to *ACTIN2* from three independent experiments. Asterisks indicate significant differences of flg22 compared with mock samples (Student’s *t* test, ^***^P < 0.001). D, Representative photographs of the 4-week-old *A. thaliana* accessions chosen for RNA-seq. E–G, 12-day-old *A. thaliana* seedlings were treated with mock or 1 μM flg22 for 1 h and extracted RNA was subjected to RNA-seq. The analysis was limited to the list of 17,856 genes showing 1:1 orthology in all tested Brassicaceae species to directly compare inter- and intra-species variation in transcriptome responses. DEGs were defined using the following criteria: q-value <0.01 and |log_2_ fold change| >1. E, Bars represent the number of up- or down-regulated DEGs in each *A. thaliana* accession. F, A Venn diagram showing the number of shared and specific DEGs in *A. thaliana* accessions. G, Heatmap of DEGs in at least one accession clustered by k-means (*k* = 15). Log_2_ expression changes compared with mock are shown. See Supplemental Data Set S5 for a list of individual genes. H, Five-week-old plants were syringe-infiltrated with mock or 1 μM flg22 24 h prior to infiltration with *Pto* DC3000 (OD_600_ = 0.0002). The log_2_ ratio of copy numbers of a bacterial gene (*oprF*) and a plant gene (*ACTIN2*) was determined by qPCR and used to represent relative *Pto* DC3000 growth. Bars represent means and SEs from two independent experiments each with three biological replicates (*n* = 6). The biological replicates from two independent experiments are represented by dots and triangles. Different letters indicate significant differences (mixed linear model, adjusted *P* < 0.01).

The observed differences in gene expression patterns point to diversification processes that might have occurred along the evolutionary trajectory of Brassicaceae plants. Alternatively, such variation in transcriptome responses can arise within a single species. To address this question, we analyzed the variation in flg22 responses among *A. thaliana* accessions. First, we tested the responsiveness of 24 *A. thaliana* accessions to flg22 using a MAPK phosphorylation assay. Flg22 treatment-induced MAPK phosphorylation in all accessions except Cvi-0, which lacks a functional FLS2 receptor, therefore representing a natural negative control (Dunning et al., 2007; Figure 4, A). To avoid underestimating the diversity in flg22 responses within *A. thaliana*, we selected 12 out of the 24 accessions that belong to distinct genetic groups (based on admixture groups from 1001genomes.org) and are geographically distributed over the United States, Europe, and Asia (Figure 4, B). We found that the expression of the early immune marker gene *PROPEP3* was induced in all 12 accessions at 1 h after flg22 treatment (Figure 4, C). We generated and analyzed the transcriptomes of five of these 12 accessions at 1 h after flg22 or mock treatment using RNA-seq. Importantly, these accessions were collected from geographically distant regions, were genetically diverse, and showed variable growth phenotypes (Figure 4, B and D). We mapped the RNA-seq reads to the *A. thaliana* Col-0 reference genome and used the same set of 17,856 1:1 orthologous genes that we used in the comparison between Brassicaceae species to avoid overestimating conservation attributable to the larger number of shared genes among *A. thaliana* accessions.

The transcriptome responses of the selected *A. thaliana* accessions to 1 h-flg22 treatment were similar in magnitude to those of other Brassicaceae species and the *A. thaliana* Col-0 accession (4,964–2,861 DEGs), ranging from 4,372 (Kon) to 2,443 (Kn-0) DEGs (Figure 4, E). However, the overlap of DEGs among the five *A. thaliana* accessions was greater than that of the four Brassicaceae species, as 1,232 DEGs (26% of the total) were shared among these five accessions, while 764 DEGs (15.7% of the total at 1 h) were shared among the four Brassicaceae species (Figure 4, F andSupplemental Figure S2). Consistent with these findings, the expression patterns of all 4,733 DEGs (differentially expressed in at least one accession) were highly conserved among the five accessions, and we did not find accession-specific expression clusters with the same clustering threshold used in the interspecies comparisons (Figure 4, G andSupplemental Data Set S5). Mapping the RNA-seq reads to the Col-0 reference genome could potentially bias the analysis toward similar expression patterns among *A. thaliana* accessions. To test for this possibility, we generated SNP-corrected genomes for each accession and re-mapped the RNA-seq reads from *A. thaliana* accessions to their own genomes. These two mapping procedures yielded comparable results (Supplemental Figure S5). Therefore, we used the initial mapping procedure to the Col-0 reference genome for the following analyses.

We speculated that the high similarity in flg22-induced transcriptional reprogramming observed in the *A. thaliana* accessions would lead to similar effects on flg22-triggered immunity against *Pto* DC3000. Indeed, flg22 significantly reduced *Pto* DC3000 titers in all accessions, although the bacterial growth under mock conditions differed among accessions, with lower bacterial titers in Gy-0 and Kon and higher titers in No-0 compared with Col-0 (Figure 4, H). Taken together, these results indicate that the within-species variation of early flg22-induced gene expression changes is smaller than the between-species variation, despite the wide global distribution of *A. thaliana*.

### Inter-species variation in transcriptome responses to flg22 exceeds intra-species variation and is incongruent with the phylogeny

**Figure 5:**
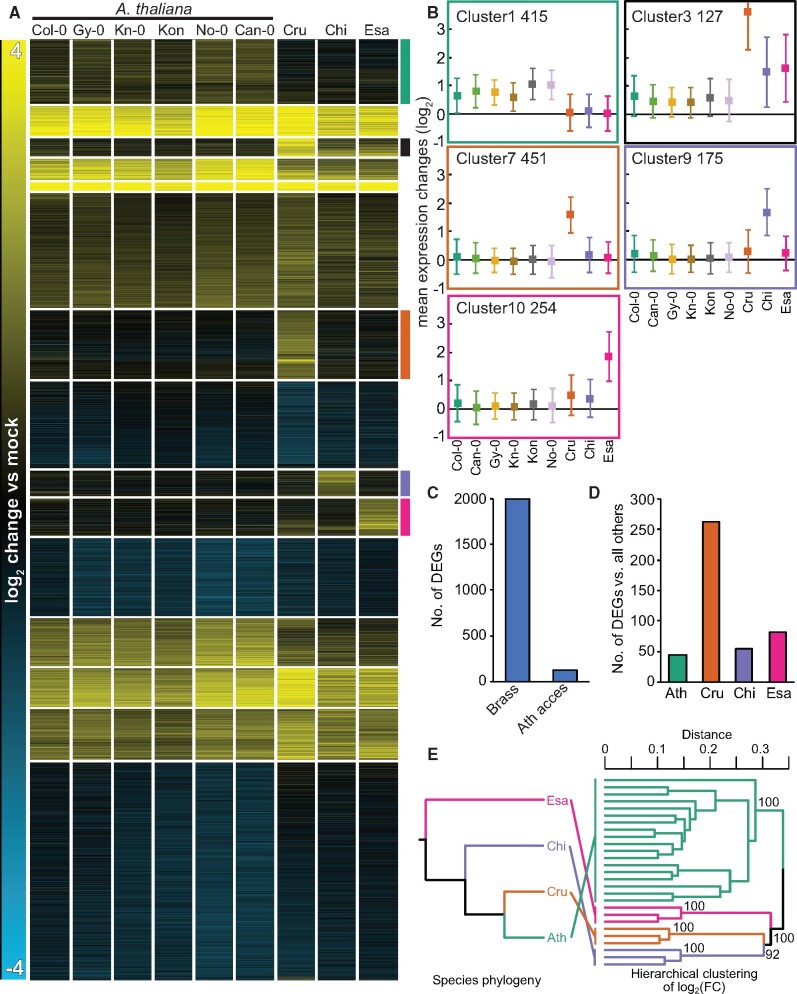
Inter-species variation exceeds intra-species variation in transcriptome responses to flg22 and is incongruent with the phylogeny. A, Log_2_ expression changes of all 5,961 DEGs 1 h after 1 μM flg22 treatment were clustered using *k*-mean clustering (*k* = 15). 1:1 orthologous genes that are differentially expressed (*q*-value <0.01; |log_2_ fold change| >1) in at least one species or accession were used. Species-specific expression clusters are highlighted by colored bars on the right side of the heatmap (Ath (green), non-Ath (black), Cru (orange) Chi (purple), Esa (magenta)). Cru, *C. rubella* (N22697); Chi, *C. hirsuta* (Oxford); Esa, *E. salsugineum* (Shandong). See Supplemental Data Set S6 for a list of individual genes. B, Mean expression changes ± SD of species-specific expression clusters in (A). The number of genes within each cluster is represented by the numbers on the top left side of each plot. C, The total number of genes that respond to flg22 significantly differently across Brassicaceae species including *A. thaliana* Col-0 (Brass) or across *A. thaliana* accessions (Ath access). *q*-value <0.01; |log_2_ fold change| >1 criteria were used. D, The number of genes that respond to flg22 significantly differently in each Brassicaceae species compared with the other three Brassicaceae species. *q*-value <0.01; |log_2_ fold change| >1 criteria were used. Ath, *A. thaliana* (Col-0). E, A hierarchical clustering with log_2_ fold changes of 1:1 orthologs. The topology of the dendrogram (right) is compared to the species tree (left). Pairwise distance matrix (1 – Pearson’s correlation coefficient) were analyzed with the R package “pvclust” with default settings. Support values for clades were obtained by 1,000 bootstrapping.

To directly compare variation in transcriptome responses to flg22 across the Brassicaceae species and within *A. thaliana*, we re-analyzed the data from all 1 h samples together. We normalized the data, identified the DEGs, and clustered the log_2_ expression changes of all 5,961 DEGs together. Similar to the previous analyses, the heatmap revealed gene clusters with species-specific signatures for each species but not a single gene cluster with *A. thaliana* accession-specific signatures (Figure 5, A and B andSupplemental Data Set S6). Wrongly assigned orthologous pairs could lead to spurious identification of species-specific expression patterns. Defining true orthologous genes between different species is challenging, especially for gene families with many homologous genes. We reasoned that if the identification of genes with species-specific expression signatures resulted from the incorrect assignment of orthologs, the species-specific gene clusters should be associated with larger gene families compared with other gene clusters. However, we did not observe such a relationship (Supplemental Figure S6, A). Therefore, the incorrect assignment of orthologous gene pairs unlikely explains the majority of the species-specific gene expression patterns. Another possibility is that distinct expression changes for genes showing species-specific patterns might be caused by differences in the basal expression level (in mock) among different Brassicaceae plants. Nevertheless, we did not find any consistent patterns in the basal expression levels of genes that would explain species-specific induction by flg22 (Supplemental Figure S6, B). Thus, inter-species variation in transcriptome responses to flg22 among the selected Brassicaceae species clearly exceeds intra-species variation among *A. thaliana* accessions.

To provide statistical support for this conclusion, we determined the number of genes that responded differently to flg22 among the Brassicaceae plants including *A. thaliana* Col-0 or among the five *A. thaliana* accessions. We detected 1,992 DEGs in the inter-species comparison and only 131 DEGs in the comparison among *A. thaliana* accessions (Figure 5, C). Of these 131 genes, only the Can-0 accession harbored one gene that responded differently compared with all other accessions. Among Brassicaceae plants, a considerable number of genes were specifically differentially expressed in only one of the species (Figure 5, D).

The observed divergent gene expression patterns between different species together with the low variation within species could have been shaped by neutral evolution or lineage-specific non-neutral evolution, such as stabilizing selection and adaptive evolution. If the transcriptome variation among Brassicaceae species was caused by stochastic processes and was thus selectively neutral, such variation should correlate with the phylogenetic distance between the species (Broadley et al., 2008). To test this possibility, we performed a hierarchical clustering of the log_2_ fold changes of all 1:1 orthologs, including both DEGs and non DEGs, at 1 h after flg22 treatment. Species-specific clades were successfully recovered with high support (bootstrap value = 100), and the relationship among the species-specific clades did not concur with the species phylogeny (Figure 5, E). These results suggest that species-specific transcriptome responses to flg22 reflect non-neutral processes during Brassicaceae evolution.

To further explore this possibility, we performed multi-optima phylogenetic Ornstein–Uhlenbeck (OU) modeling (Hansen, 1997) for each ortholog using log_2_ fold changes as trait values to be fit. The hierarchical clustering results could be strongly affected by genes with large expression variation between species, potentially providing a distorted view of the evolution of gene expression. However, because multi-optima phylogenetic OU modeling is a gene-by-gene analysis in which each gene contributes to the overall patterns equally, the results are expected to be robust against biases from a small number of genes with large effects. In these models, the strength of neutral drift and the pull toward the estimated theoretical optimum were taken into account with parameters *σ*^2^ and *α*, respectively. Potentially adaptive changes were searched as regime shifts of the theoretical optimum by the phylogenetic LASSO algorithm with a phylogeny-aware information criterion (pBIC) (Khabbazian et al., 2016). We note that the necessarily limited number of species that were sampled in this study might inflate false positives. The regime shifts were detected in 3,136 out of 5,961 orthologous genes, suggesting frequent evolutionary changes in flg22-triggered transcriptional responses that could potentially be selectively driven (Supplemental Figure S7). Notably, the four species-specific clusters (Figure 5, A) showed the highest shift frequencies in the branches connected to the corresponding species compared with the others (Supplemental Figure S7). These results suggest that these clusters are enriched in a group of genes that could have evolved through the non-neutral switching of selective regimes in addition to neutral drift and/or stabilizing selection.

**Figure 6:**
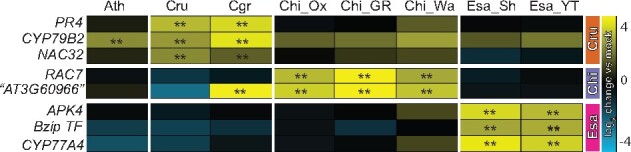
Species–specific expression signatures are conserved in sister species and accessions. Expression of selected genes showing species–specific expression signatures in Figure 5, A were determined in available sister species and accessions by RT-qPCR. Gene expression was normalized to *ACTIN2*. The colored bars on the right indicate genes showing Cru- (orange), Chi- (purple), or Esa- (magenta) specific expression signatures. The heatmap represents mean log_2_ changes of flg22 samples compared with mock from three independent experiments, each with two biological replicates (*n* = 6). Asterisks indicate significant flg22 effects (mixed linear model, *P* < 0.01). Ath, *A. thaliana* Col-0; Cru, *C. rubella*; Cgr, *C. grandiflora*; Chi_Ox, Chi_GR, Chi_Wa, different *C. hirsuta* accessions; Esa_Sh, *E. salsugineum* Shandong; Esa_YT, Esa Yukon.

The conservation of gene induction across six *A. thaliana* accessions (Figure 5, A and B; Cluster 1) suggests that the observed species-specific expression signatures in Brassicaceae species might represent novel inventions in the respective species. To test this idea, we measured changes in the expression of selected genes showing species-specific expression signatures in different accessions or sister species of *C. rubella*, *C. hirsuta*, and *E. salsugineum* by RT-qPCR. For this, we selected grand shepherd’s-purse (*Capsella grandiflora*; Cgr, a sister species of *C. rubella*), two additional *C. hirsuta* accessions (Wa and GR2), and one additional *E. salsugineum* accession (YT). We selected *PR4*, *CYP79B2*, and *NAC32* as *C. rubella*-specific genes. *PR4* and *NAC32* were specifically induced in both *C. rubella* and *C. grandiflora*, while *CYP79B2* was induced in these two species as well as *A. thaliana* Col-0 (Figure 6). The two *C. hirsuta*-specific genes *RAC7* and *AT3G60966* (as there is no common name for *AT3G60966*, we used the *A. thaliana* gene code) were specifically induced in all three *C. hirsuta* accessions, with the exception of *AT3G60966*, which was also induced in *C. grandiflora*. All three *E. salsugineum*-specific genes (*APK4*, *bZip TF*, and *CYP77A4*) were induced only in *E. salsugineum* accessions (Figure 6). Together, these findings demonstrate that the specific patterns of gene expression observed in each of the tested Brassicaceae are conserved features in the respective species or lineage.

### WRKY TFs are central for flg22-triggered gene induction and may be responsible for the emergence of species-specific gene induction

**Figure 7:**
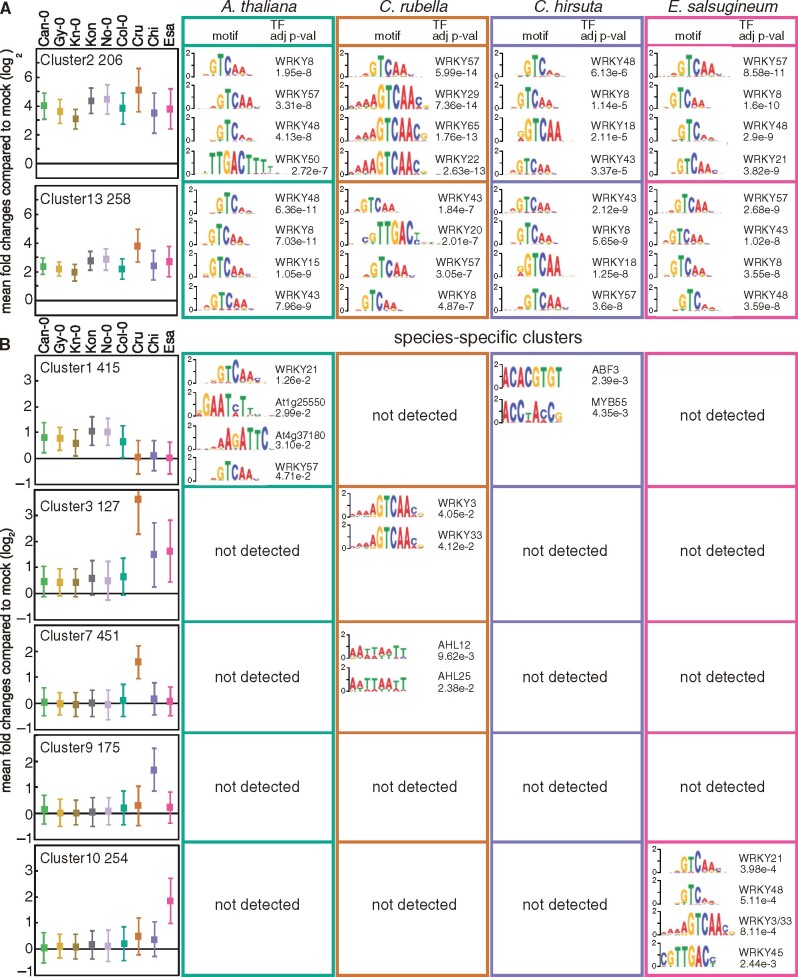
Enrichment of known TF-binding motifs in the 5′-regulatory regions of genes in shared and species-specific clusters. The 500 bp upstream sequences of the transcription start sites of the genes in the individual clusters were tested for enrichment of known TF binding motifs. Names of TFs, sequence logos, and adjusted *P*-values (up to the top four) of motifs are shown for each Brassicaceae species. The names of clusters, the number of DEGs, and mean log_2_ fold changes ± SD compared with mock are shown on the left side. See Supplemental Data Set S7 for the other clusters. For the complete list of all enriched TF binding motifs, please see Supplemental Data Set S7. Ath, *A. thaliana* (Col-0); Cru, *C. rubella* (N22697); Chi, *C. hirsuta* (Oxford); Esa, *E. salsugineum* (Shandong).

Changes in gene transcription are often mediated by the binding of specific TFs to 5′-regulatory regions (Baxter et al., 2012). However, our understanding of how gene expression is regulated during PTI, whether gene regulatory mechanisms differ in different species, and how a given species acquires a new mode of gene regulation is far from complete. Together with genomic resources, we reasoned that our datasets, which revealed both conserved and diversified gene expression patterns in the Brassicaceae species, may provide valuable insights into these questions. To this end, we searched the 5′-regulatory regions (500 bp upstream of the transcriptional start site) of the genes in each of the 15 gene clusters (Figure 5) for known TF-binding motifs in each Brassicaceae species. Our analysis revealed that multiple motifs, which are typically bound by WRKY TFs, are highly enriched in the 5′-regulatory regions of genes in common flg22-induced clusters such as Clusters 2, 5, 13, and 14 (Figure 7, A andSupplemental Figure S8). In *A. thaliana*, WRKY TFs are known to regulate transcriptional reprogramming during plant immunity, including responses to flg22 (Tsuda and Somssich, 2015; Birkenbihl et al., 2017). Also, the WRKY gene family has significantly expanded in land plants, which was likely required for adaptation to the terrestrial environment (One Thousand Plant Transcriptomes Initiative, 2019). Our results suggest that transcriptional induction mediated by WRKY TFs is a conserved mechanism in response to flg22 across these Brassicaceae species. In addition, the 5′-gene regulatory regions of flg22-induced expression clusters in *A. thaliana*, *C. rubella*, and *C. hirsuta* (Clusters 13, 6, and 14, respectively) were significantly enriched for CAMTA TF-binding motifs (Supplemental Data Set S7), which play an important role in early immune transcriptional reprogramming (Jacob et al., 2018).

Interestingly, we found that WRKY TFs are associated with the 5′-regulatory regions of genes showing species-specific induction only in the species that are highly flg22-responsive. For instance, in Clusters 1, 3, and 10, WRKY TF-binding motifs were only enriched in 5′-regulatory regions of flg22-induced genes in *A. thaliana*, *C. rubella*, and *E. salsugineum*, respectively (Figure 7, B). In addition, in the *C. rubella*-specific expression cluster (Cluster 7), AHL TF-motifs were enriched only in *C. rubella* 5′-regulatory regions (Figure 7, B). AHL TFs have been associated with plant developmental processes, but some AHL TFs are involved in MAMP-induced gene expression (Lou et al., 2014; Mine et al., 2018). These results suggest that in these Brassicaceae plants, the emergence of cis-regulatory sequences that are bound by specific TFs (such as WRKY TFs) facilitated the evolution of distinct gene induction patterns.

### Variation in coding sequences shows no strong correlation with transcriptome variation

**Figure 8:**
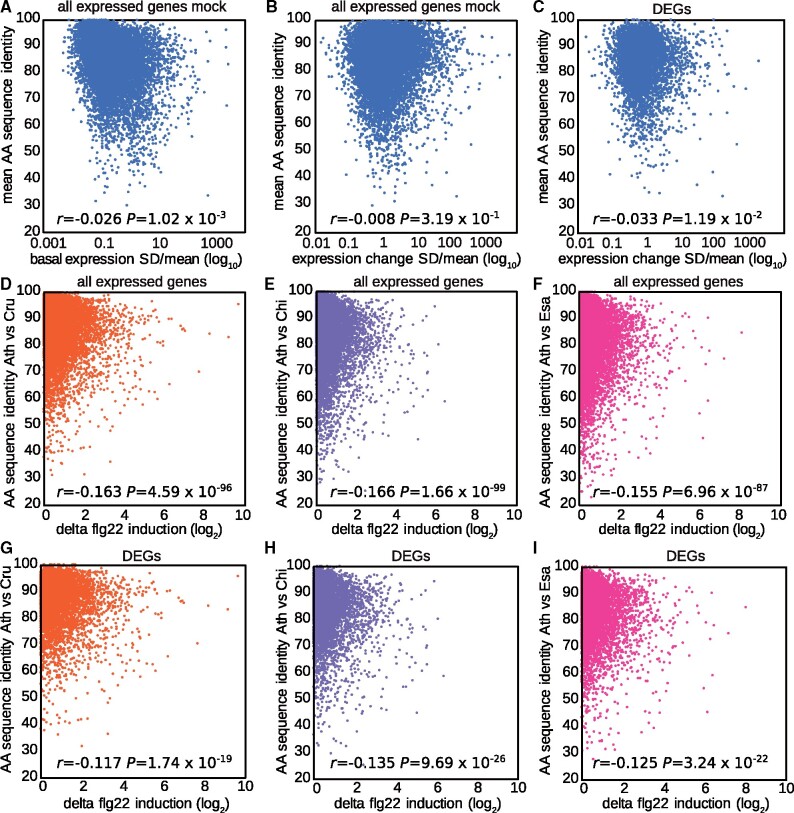
Gene expression variation does not correlate with coding sequence variation. A, Mean AA sequence identities of *C. rubella*, *C. hirsuta*, and *E. salsugineum* to *A. thaliana* (*y*-axis) were plotted against the SD/mean of the expression values in mock samples of all four Brassicaceae plants for all expressed genes (*x*-axis). B and C, Mean AA identities of *C. rubella*, *C. hirsuta*, and *E. salsugineum* to *A. thaliana* were plotted against the SD/mean of flg22-induced expression changes in all four Brassicaceae plants for all expressed genes with 1:1 orthologs (16,100 genes) (B) or 5,961 DEGs (C). D–I, Pairwise AA sequence identities of *C. rubella* (D, G), *C. hirsuta* (E, H) and *E. salsugineum* (F, I) to *A. thaliana* were plotted against the flg22-induced expression changes between the compared species for all expressed genes (D–F) or DEGs (G–I).

Previous studies reported a positive correlation between gene expression and coding sequence evolution and suggested that similar selective forces might have acted on both modes of evolution (Khaitovich et al., 2005; Slotte et al., 2011; Hunt et al., 2013; Hodgins et al., 2016), although it should be noted that in some studies, this correlation was organ-dependent or not detected at all (Tirosh and Barkai, 2008; Whittle et al., 2014). Thus, the relationship between gene expression and coding sequence evolution appears to be species- or condition-dependent. Therefore, we asked whether the variation in basal or flg22-triggered expression changes is correlated with variation in amino acid (AA) sequences among the tested Brassicaceae species. We compared the standard deviation divided by the mean of the expression levels in mock-treated RNA-seq samples (1 h) of *A. thaliana* Col-0 and other Brassicaceae plants with the mean AA sequence identities between *A. thaliana* Col-0 and each of the other species. We found no correlation between the variation in AA sequence and basal gene expression (Figure 8, A). Similarly, we compared flg22-induced expression changes of all expressed genes or DEGs (1 h) with AA sequence identities and found no correlation (Figure 8, B and C). Finally, we tested whether pairwise differences in flg22-induced expression changes between *A. thaliana* and individual Brassicaceae species were linked to AA sequence diversification. Again, we did not find any strong correlation (Figure 8, D–I). We also tested for synonymous or non-synonymous polymorphism (dN/dS) and did not find any strong correlation (Supplemental Figure S9). Thus, variation in gene expression at both the basal level and in response to flg22 does not correlate with variation in AA sequences or dN/dS, suggesting that different selective forces influence gene expression and coding sequence evolution in these Brassicaceae species.

### Purifying selection may have acted on the regulatory regions of conserved flg22-responsive genes across Brassicaceae species

To test whether genes displaying a specific response in *A. thaliana* (Cluster 1) may have been subjected to recent adaptive pressures, we compared their patterns of polymorphism and divergence at upstream and coding regions with other gene clusters displaying no *A. thaliana*-specific expression (Clusters 2, 5, 7, and 9). If recent and recurrent regulatory adaptive mutations in *A. thaliana* were the ultimate cause of the expression specificity observed in Cluster 1, we should observe an elevated divergence between *A. thaliana* and *Arabidopsis lyrata* at regulatory regions compared with the other clusters and potentially a distribution of allele frequencies skewed toward higher frequency classes compared with neutral expectations (Nielsen, 2005). Instead, our results indicate that the genetic variation observed in genes with *A. thaliana*-specific responses is overall in line with the variation observed in other clusters regardless of species-specificity (Supplemental Figure S10). However, Cluster 5 (highly induced in all species) showed the lowest genetic divergence in its upstream regions (the first 100 bp upstream of the gene), while the neutral synonymous variation for the same cluster was the highest (Supplemental Figure S10). This suggests that this lower divergence at upstream regions is not the result of lower mutation rates but rather the result of stronger purifying selection acting on the regulatory regions of those genes with conserved expression patterns in this cluster.

### Differences in metabolome profiles in response to flg22 among the Brassicaceae species

**Figure 9:**
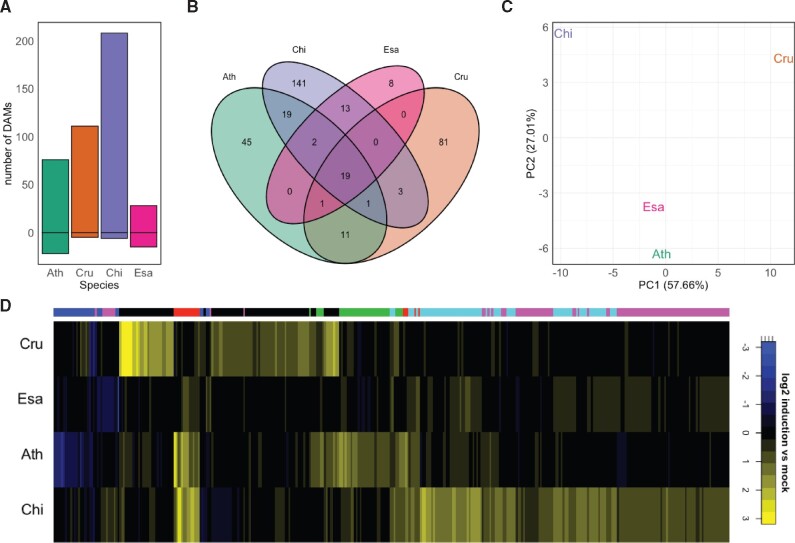
Flg22 triggers unique metabolomic changes in the Brassicaceae species examined. Metabolite profiles were analyzed by HPLC–MS 24 h after mock or flg22 treatment in 12-day-old Brassicaceae seedlings. A, DAMs were determined using the following criteria: treatment effect or interaction treatment × species was significant with *q*-value <0.05, |log_2_ fold change| >0.585 and significance of the difference between treatment and control with *P* < 0.05. The bars represent the number of up- or down-regulated DAMs in each species. Ath, *A. thaliana* (Col-0); Cru, *C. rubella* (N22697); Chi, *C. hirsuta* (Oxford); Esa, *E. salsugineum* (Shandong). B, A Venn diagram showing the number of shared and unique DAMs between species. All DAMs present in at least one species were used. C, Principal component analysis of DAMs in at least one species. D, Heatmap of log_2_ fold changes for DAMs in at least one species clustered by *k*-means clustering (*k* = 6; clusters marked in the color bar on top of the heatmap).

Some genes showing species-specific expression patterns were associated with GO terms connected to secondary metabolism (Supplemental Data Set S8). This prompted us to investigate whether flg22 treatment differentially affects the metabolite profiles of Brassicaceae plants. In unbiased HPLC–MS analysis, we detected various differentially accumulating metabolites (DAMs; *q*-value <0.05, minimum fold change of 1.5) in response to flg22 among the four Brassicaceae plants (Figure 9, A). Interestingly, most flg22-induced changes in metabolite accumulation were species-specific, and only 19 out of 360 DAM signals were commonly affected by flg22 in all tested Brassicaceae species, indicating a strong diversification of the native metabolome and its reprogramming in response to flg22 (Figure 9, B). This notion was further supported by the clustering of log_2_ fold changes for all DAMs, which showed only a limited number of overlaps between metabolome alterations, as well as by principal component analysis (Figure 9, C and D).

## Discussion

Our comparison of transcriptome responses within *A. thaliana* accessions and among four Brassicaceae species revealed fundamental features of transcriptome evolution: we identified conserved core genes and species-specific responsive genes across Brassicaceae plants during flg22-induced PTI. A core set of responsive genes conserved across Brassicaceae likely reflect important gene regulatory processes in PTI, whereas each species evolved its specific responses for species-specific arms. Our unified experimental setup for all transcriptome comparisons has allowed us to detect gene expression variation among these species.

We have shown that transcriptome responses to flg22 are remarkably conserved within *A. thaliana*. A previous study found that variation in the expression of stress-responsive genes accounted for the majority of divergence in expression among *A. thaliana* accessions (Kawakatsu et al., 2016), and many studies have described the strong plasticity of immune-responsive gene expression in the face of other environmental perturbations such as abiotic stress (Singh et al., 2014; Ueno et al., 2015; Coolen et al., 2016; Berens et al., 2019). Since the *A. thaliana* accessions that we examined were collected from habitats with distinct climatic conditions (such as the Canary Islands and Lithuania) and have diverse genetic backgrounds, these highly conserved early transcriptome responses to flg22 were surprising. This finding indicates that short-term evolution in divergent environments did not introduce major variation in early transcriptional responses during PTI.

We found that the expression changes of large numbers of genes are conserved among different Brassicaceae species during flg22-induced PTI. Many of these genes have not been previously characterized or linked to plant defense. Thus, our dataset provides the basis and rationale for future studies. In addition, the conservation of transcriptome responses to flg22 over 26 million years of Brassicaceae evolution suggests the functional importance of these as-yet-uncharacterized genes. Many studies have demonstrated the importance of TFs that regulate the expression of specific genes during PTI (Birkenbihl et al., 2017; Jacob et al., 2018). However, the lack of a method to efficiently and specifically block transcriptional reprogramming during PTI means that it remains obscure whether genome-wide transcriptional reprogramming during PTI is required for plant defense against pathogens and/or for adaptation to their environments. The evolutionary constraint feature in the transcriptome responses strongly suggests that the massive transcriptional reprogramming during PTI is advantageous for Brassicaceae plants in nature.

While all tested Brassicaceae species deployed rapid, massive transcriptional reprogramming as well as MAPK activation, they exhibited different PTI outputs. For instance, in *C. hirsuta* and *E. salsugineum*, the flg22-elicited transcriptional response was not associated with flg22-induced resistance against *Pto* DC3000*.* This seems counterintuitive for species not benefitting from costly transcriptional reprogramming. Maintaining transcriptional reprogramming during PTI is analogous to the retention of susceptible alleles for *Rps2*, a resistance gene for the pathogen virulence effector AvrRpt2 (MacQueen et al., 2016). One possible explanation is that susceptible *Rps2* alleles encode recognition specificities for pathogen effectors that have yet to be identified (MacQueen et al., 2016). Similarly, transcriptional reprogramming induced by flg22 may be associated with effective resistance against different bacterial pathogens or the control of plant microbiota in *C. hirsuta* and *E. salsugineum* (Hacquard et al., 2017; Chen et al., 2020). Understanding how diversity in PTI responses across plant species is linked to plant adaptation would be crucial for comprehending the role of PTI.

In addition to conserved transcriptome responses, each of the tested Brassicaceae species exhibited species/lineage-specific expression signatures during flg22-induced PTI. We have shown that species-specific expression patterns are conserved among multiple accessions or sister species in the respective species. Moreover, variation in transcriptome responses during flg22-induced PTI was incongruent with the Brassicaceae phylogeny, which is inconsistent with the notion that variation was caused solely by genetic drift. Thus, some of the species-specific expression signatures observed in this study during flg22-induced PTI may be selectively driven expression shifts.

We have also shown that interspecific differences are larger than intraspecific variations in the early transcriptome response during PTI. This expression divergence could be explained by variation in the expression and function of TFs and/or variation in cis-regulatory elements in 5′-gene regulatory regions that coincide in a lineage-specific manner. It has been thought that most intraspecific variations could be attributed to cis-associated differences that are tightly constrained by linkage disequilibrium, while interspecific differences largely occur because trans-associated alterations are a larger mutational target (Signor and Nuzhdin, 2018). However, disentangling the contributions of trans- and cis-components of transcriptional control remains challenging. A recent study revealed that transcriptome variation is disposed to strong selection pressure in perturbed environments, in particular, genes with expression stochasticity and plasticity (Groen et al., 2020). In line with the important role of WRKY TFs in gene induction during immunity (Tsuda and Somssich, 2015; Birkenbihl et al., 2017), we have revealed that WRKY-TF binding motifs are highly enriched in the 5′-gene regulatory sequences of species in which the genes are induced. This suggests that some specific gains of TF-binding motifs in the 5′-gene regulatory regions account for the evolution of some species-specific flg22-responsive expression changes. It is also possible that duplicated genes could be responsible for species-specific differences that we may have missed in this study, as we entirely omitted lineage-specific duplicates. However, this would not introduce a very serious bias, as a large proportion of genes in the genomes (17,856) were successfully analyzed in this study.

Whether gene expression evolution correlates with coding sequence evolution remains a contentious topic (Tirosh and Barkai, 2008). Some studies found a positive correlation between gene expression and coding sequence evolution and argued that similar selection pressures act on both modes of evolution (Hunt et al., 2013; Whittle et al., 2014). In contrast, others have concluded that gene expression evolution may provide additional evolutionary capacity if the sequence of the respective gene is under evolutionary constraint (Shapiro et al., 2004; Harrison et al., 2012; Dean et al., 2015). In this scenario, gene expression variation would not be correlated with coding sequence evolution (Tirosh and Barkai, 2008; Renaut et al., 2012; Uebbing et al., 2016). In the current study, we found almost no correlation between variation in basal gene expression or flg22-induced gene expression changes and variation in their AA sequences and dN/dS. The connection between gene expression and coding sequence variation might depend on the species and growth conditions (Whittle et al., 2014). Further studies, especially in the plant field, are needed to better define the relationship between these two modes of evolution.

## Methods

### Plant materials

Plant materials used in this study are described in Tables 1–3.

**Table 1:** Brassicaceae species and accessions used in this study

Species	Accession	Abbreviation	Reference
***A. thaliana***	**Col-0**	Ath	Kenichi Tsuda lab
***C. rubella***	**N22697**	Cru	Slotte et al. (2013)
*C. grandiflora*	Unknown	Cgr	Slotte et al. (2013)
***C. hirsuta***	**Oxford**	Chi	Hay and Tsiantis (2006)
*C. hirsuta*	Wa	Wa	Cartolano et al. (2015)
*C. hirsuta*	GR2	GR2	Miltos Tsiantis lab
***E. salsugineum***	**Shandong**	Esa	Miltos Tsiantis lab
*E. salsugineum*	Yukon	YT	Miltos Tsiantis lab

Bold entries indicate species used for RNA-seq and metabolome analyses.

**Table 2:** *A. thaliana* accessions used in this study

Accession	Cs Number	Country	Admixture Group^a^	Reference
An-1	CS76435	BEL	Admixed	1001 Genomes Consortium (2016)
Bla-1	CS76451	ESP	Spain	1001 Genomes Consortium (2016)
**Can-0**	**CS76740**	ESP	Relict	1001 Genomes Consortium (2016)
**Col-0**	**CS76778**	USA	Germany	1001 Genomes Consortium (2016)
CVI-0	CS76789	CPV	Relict	1001 Genomes Consortium (2016)
Edi-0	CS76831	UK	Admixed	1001 Genomes Consortium (2016)
**Gy-0**	**CS78901**	FRA	Western Europe	1001 Genomes Consortium (2016)
HR10	CS76940	UK	Western_Europe	1001 Genomes Consortium (2016)
Kas-2	CS78905	IND	Asia	1001 Genomes Consortium (2016)
**Kn-0**	**CS76969**	LTU	Central_Europe	1001 Genomes Consortium (2016)
**Kondara**	**CS76532**	TJK	Asia	1001 Genomes Consortium (2016)
Ms-0	CS76555	RUS	Asia	1001 Genomes Consortium (2016)
**No-0**	**CS77128**	GER	Central_Europe	1001 Genomes Consortium (2016)
Pna-17	CS76575	USA	Germany	1001 Genomes Consortium (2016)
Rsch4	CS77222	RUS	Germany	1001 Genomes Consortium (2016)
Se-0	CS76597	ESP	Spain	1001 Genomes Consortium (2016)
Sf-2	CS77247	ESP	Spain	1001 Genomes Consortium (2016)
Sorbo	CS78917	TJK	Asia	1001 Genomes Consortium (2016)
Tamm-27	CS77341	FIN	North_Sweden	1001 Genomes Consortium (2016)
Ts-1	CS76615	ESP	Spain	1001 Genomes Consortium (2016)
Tsu-0	CS77389	JPN	Admixed	1001 Genomes Consortium (2016)
Van-0	CS76623	CAN	Western_Europe	1001 Genomes Consortium (2016)
Wil-2	CS78856	LTU	Central_Europe	1001 Genomes Consortium (2016)
Wu-0	CS78858	GER	Germany	1001 Genomes Consortium (2016)

Bold entries indicate accessions used for RNA-seq. ^a^Admixture group (1001 Genomes Consortium, 2016).

**Table 3:** *A. thaliana* mutants used in this study

Species	Mutant allele	Locus	Source
*A. thaliana*	*sid2-2*	AT1G74710	Tsuda et al. (2008)
*A. thaliana*	*fls2* (SAIL_691C4)	AT5G46330	Zipfel et al. (2004)

### Plant growth

Seeds were sterilized by vortexing in 70% ethanol for 5 min, followed by 6% NaClO for 10 min, washed five times with sterile water, and stratified in sterile water at 4°C for 5–7 days. Sterilized seeds were grown on half Murashige and Skoog (MS) Agar (2.45 g/L M&S+Vitamins+MES (Duchefa, , Haarlem, Netherlands), 1% sucrose, 0.5% plant agar, pH 5.8) plates in a Percival plant growth chamber (CU-36LX5D, Percival, USA) at 22°C with 10 h of light (white fluorescent lamps) for 11 days if not stated otherwise. Eleven-day-old seedlings were transferred to liquid half MS Medium (2.45 g/L M&S + Vitamins + MES (Duchefa), 1% sucrose) 1 day before flg22 treatment. Alternatively, 12-day-old seedlings were transferred to soil (Stender, Schermbeck, Germany) and grown at 23°C/20°C under a 10/14 h (light/dark) cycle and 60% relative humidity. Soil-grown plants were transferred to another chamber at 22°C with a 12 h photoperiod and 60% relative humidity 3 days before bacterial inoculation.

### Flg22 treatment

Eleven-day-old seedlings were transferred from half MS Agar to 24-well plates, each containing 1.6 mL of half MS Medium, for 24 h prior to treatment. If not otherwise stated, 5–10 seedlings per sample were transferred to each well. For the flg22 treatment, 800 µL of 3 µM flg22 (EZBiolab Inc., Carmel, IN, USA) solution was added to the medium containing the seedlings, resulting in a final concentration of 1 µM flg22. Seedlings were harvested in liquid nitrogen at the indicated time points, and three wells were combined into one biological sample. The samples were stored at −80°C until use.

### Seedling growth inhibition assay

Seven-day-old seedlings grown on half MS Agar were transferred to 1.6 mL of half MS Medium with or without 1 µM flg22 and grown for another 12 days under these conditions. The fresh weight of 12 pooled seedlings was then measured. The experiment was carried out three independent times, and statistical analysis was performed with log_2_-transformed fresh weight values.

### Bacterial growth assay

To prepare bacterial inoculum, *P. syringae* pv. *tomato* DC3000 (*Pto* DC3000) or the T3SS deficient *Pto* DC3000 mutant *Pto hrcC* (Tsuda et al., 2008) was grown on NYGA agar (2% glycerol, 0.5% Bacto Peptone, 0.3% yeast extract, and 1% Bacto Agar, pH 7.0) plates containing 25 µg/mL rifampicin for 3 days at 28°C. The bacterial strains were transferred to liquid NYGA medium containing 25 µg/mL rifampicin and incubated overnight at 28°C with shaking at 200 rpm to a final OD_600_ between 0.8 and 1. The bacteria were pelleted by centrifugation at 5,000 rpm and washed twice with sterile 5 mM MgSO_4_ before dilution to an OD600 of 0.0002 (*Pto* DC3000) or 0.001 (*Pto hrcC*).

Four to five-week-old plants were used. Two leaves per plant were infiltrated with 1 µM flg22 or sterile water (mock) using a needleless syringe. One day later, leaves treated with flg22 or mock solution were infiltrated in the early afternoon with the bacterial suspension. Two days after bacterial infiltration, two leaf disks (0.565 cm^2^) per sample from two leaves were crushed in 400 µL sterile MgSO_4_ using a Retsch mixer mill. Dilution series were made and streaked on NYGA agar plates containing 25 µg/mL rifampicin. The plates were incubated for 2 days at 28°C before colony forming units were counted.

Alternatively, bacterial growth was quantified using a qPCR-based method as previously described (Ross and Somssich, 2016). In brief, DNA was extracted from bacteria-infiltrated leaves using a FastDNA Spin Kit (MP Biomedicals, Santa Ana, CA, USA). Extracted DNA was quantified and adjusted to 8.75 µg/µL to achieve a final concentration of 35 µg DNA in a qPCR. Bacterial DNA was quantified using the expression levels of the Pto DC3000-specific *oprF* gene relative to plant *ACTIN2* (*ACT2*) DNA. ΔCt values were calculated by subtracting the Ct value of the target gene from that of *ACT2*. These ΔCt values were considered to be log_2_ values and used for statistical analysis. The primers used are listed in Supplemental Data Set S9.

### MAPK phosphorylation assay

The MAPK phosphorylation assay was performed as previously described (Tsuda et al., 2009). In short, 12-day-old seedlings were treated with 1 µM flg22 or mock for 15 min, frozen in liquid nitrogen, and ground with four metal beads in a Retsch MM 400 mixing mill (Retsch, Haan, Germany). Then, 150 µL of MAPK extraction buffer (50 mM Tris–HCL [pH 7.5], 5 mM EDTA, 5 mM EGTA, 2 mM DTT, 10 mM NaF, 50 mM β-glycerolphosphate, 10% glycerol, complete proteinase inhibitor and PhosSTOP phosphatase inhibitor [both from Roche, Germany]) was added to the sample, and protein was extracted by centrifugation (4°C, 12,000 rpm). Protein concentration was determined using a Coomassie Protein Assay Kit (Thermo Fisher Scientific, Waltham, MA, USA), and 25 µg of protein was separated by SDS-PAGE for 1 h at 100 V. MAPK phosphorylation was detected via immunoblotting using an antiphospho-p44/42 MAPK antibody (dilution 1:5,000 in TBST; Cell Signaling Technology, Danvers, MA, USA) as primary and HRP-conjugated anti-rabbit IgG (1:10,000 in TBST; Sigma–Aldrich, St. Louis, MO, USA) as secondary antibody. Luminescence was detected using SuperSignal West Femto Chemiluminescent Reagent (Thermo Fisher Scientific) and a ChemiDoc MP imaging system (Bio-Rad, Hercules, CA, USA).

### RNA extraction, cDNA synthesis, and RT-qPCR

Seedling samples were ground in 2-mL reaction tubes with four metal beads using a Retsch MM 400 mixing mill (Retsch). RNA was extracted using peqGOLD TriFast with an additional DNA digestion step using DNAse I (Roche). The RNA was precipitated overnight at 4°C in 100% ethanol containing 115 mM Na–Ac (pH 5.2; Sigma–Aldrich, Munich, Germany) to further clean up and increase RNA yield. RNA quality and quantity were determined using a NanoDrop spectrophotometer (Thermo Fisher Scientific). Subsequently, cDNA was synthesized from 4 µg DNase-treated total RNA using Superscript II or IV Reverse Transcriptase (Thermo Fisher Scientific) according to the manufacturer’s instructions. qPCR was performed on a CFX Connect Real-Time PCR Detection System (Bio-Rad) using EvaGreen (Biotium, Fremont, CA, USA). The target gene was quantified relative to the expression of *ACT2* from *A. thaliana* or other Brassicaceae plants. ΔCt values were calculated by subtracting the Ct value of the target gene from that of *ACT2*. These ΔCt values were considered to be log_2_ values and were further used for statistical analysis. Primers used are listed in Supplemental Data Set S9.

### Statistical analysis

Statistical analysis for the seedling growth inhibition assay, bacterial growth assay, and RT-qPCR was performed using a mixed linear model with the function lmer implemented in the lme4 package within the R environment. To meet the assumptions of the mixed linear model, we log-transformed the raw data, when needed. The following model was fit to the data: measurementgyr ∼ GYgy + Rr + ɛgyr, with GY denoting the genotype:treatment interaction effect; R the biological replicate effect; and *ɛ* the residual. The *P*-values calculated in two-tailed *t* tests were corrected for multiple hypothesis testing using the *q*-value package when samples were compared with each other in a given figure panel.

### RNA-seq

RNA-seq experiments were independently performed three times. In each experiment, mock and flg22 treatments were conducted side by side. These three biological replicates were used for RNA-seq experiments. RNA quality was checked with the Agilent 2100 Bioanalyzer or Caliper LabChip GX device. PolyA enrichment and library preparation were performed with a NEBNext Ultra Directional RNA Library Prep Kit for Illumina (New England Biolabs, Ipswich, MA, USA). Libraries were quantified by fluorometry, immobilized, and processed onto a flow cell with a cBot (Illumina, San Diego, CA, USA), and subjected to sequencing-by-synthesis with HiSeq version 3 chemistry. Library construction and RNA sequencing were performed by the Max Planck-Genome-center Cologne (http://mpgc.mpipz.mpg.de/home/) with a single 100 bp (*A. thaliana* Col-0, *C. rubella*, *C. hirsuta*, and *E. salsugineum*) or 150 bp reads (all other *A. thaliana* accessions) using the Illumina HiSeq2500 or HiSeq3000 platform, respectively. After quality control, sequencing reads were mapped to respective reference genomes (Table 4) using TopHat2 (version 2.1.1) with default parameters, except for the parameters described in Table 5. The resulting bam files were used to count the number of reads per gene using HtSeq (version 0.6.0) software with default parameters. To exclude biases caused by mapping sequence reads of different *A. thaliana* accessions to the Col-0 genome, we created mapping genome files for each *A. thaliana* accession by correcting the Col-0 reference genome with SNP data available for these accessions. We downloaded the variants table for each accession from the website of the 1001 Genomes Project (intersection_snp_short_indel_vcf version 3.1 dataset). The pseudo-genome sequence of each accession was inferred by replacing the reference allele with the corresponding alternative allele using the pseudogeno function implemented in GEAN software. We created general feature format files by projecting the coordinates of the TAIR10 gene annotations onto the coordinates of each accession with the gffCoordinateLiftOver function of GEAN (Song et al., 2019). With these files, we performed a second mapping as described above.

**Table 4:** Reference genomes used for RNA-seq analysis

Species	Reference genome	Publication	Source
*A. thaliana*	TAIR 10	Lamesch et al. (2012)	Phytozome 10
*Ath accessions*	SNP corrected TAIR10		This study
*C. rubella*	Version 1.0	Slotte et al. (2013)	Phytozome 10
*C. hirsute*	Version 1.0	Gan et al. (2016)	http://chi.mpipz.mpg.de/
*E. salsugineum*	Version 1.0	Yang et al. (2013)	Phytozome 10

**Table 5:** TopHat2 parameters used for mapping RNA-seq reads

TopHat2 parameter	Value
−-read mismatches	10
−- read-gap-length	10
−- read-edit-dist	20
−-min-anchor-length	5
−-splice-mismatches	2
−-min-intron-length	30
−-max-intron-length	1,000
−-max-insertion-length	20
−-max-deletion-length	20
−-max-multihits	10
−-segment-mismatches	3
−-min-coverage-intron	30
−-max-coverage-intron	10,000
−-library-type	fr-firstrand
−-b2	Very sensitive

The read counts determined by HTSeq were analyzed in the R environment (version 3.3.1) using the edgeR (version 3.14.0) and limma (version 3.28.14) packages. Genes expressed at low levels were excluded from analysis by filtering out genes with a mean read count ˂10 counts per sample. Read counts then were normalized using TMM normalization embedded in the edge R package, and the data were log_2_-transformed using the voom function within the limma package to yield log_2_ counts per million. For individual analysis of Brassicaceae species and *A. thaliana* accession data, a linear model was fit to each gene using the lmFit function of limma with the following equation: Sgyr = GYgy + Rr + ɛgy, where S denotes log_2_ expression value, GY represents the genotype:treatment interaction and random factors, R indicates biological replicate, and *ɛ* represents residual. For the combined analysis of Brassicaceae species and *A. thaliana* accession data, the replicate effect was removed from the linear model, resulting in the following terms: Sgy = GYgy + ɛgy_,_ where S denotes log_2_ expression value, GY represents genotype:treatment interaction, and *ɛ* represents residual. For variance shrinkage of calculated *P*-values, the eBayes function of limma was used. The resulting *P*-values were corrected for multiple testing by calculating the false discovery rate (FDR; or *q*-value) using the *q*-value (version 2.4.2) package.

Normalization and determination of DEGs were performed separately for each Brassicaceae species and each *A. thaliana* accession. To compare expression changes mediated by flg22 between Brassicaceae plants, we used Best Reciprocal BLAST to identify genes that show a 1:1 ortholog with a corresponding *A. thaliana* gene and only kept the genes with 1:1 orthologs in every Brassicaceae species. Genes with a one-directional best hit relationship were not included in these analyses. This resulted in a set of 17,856 1:1 ortholog genes. We restricted the analysis of *A. thaliana* accessions to the same set of 17,856 genes to enable a direct comparison of results obtained from Brassicaceae and *A. thaliana* accession analysis. To directly compare Brassicaceae plants with *A. thaliana* accessions, we further normalized and determined DEGs for all 1-h samples together using the set of 17,856 orthologous genes. This approach enabled us to compare basal expression levels between Brassicaceae and *A. thaliana* accessions.

The R packages and software used for further analysis of the sequencing data are listed in Table 6. Heatmaps and *k*-mean clustering of DEGs were generated using the Genesis software with default parameters.

**Table 6:** Software and packages used in this study

Software/package	Version	Citation	Use
AME	4.12.0	McLeay and Bailey (2010)	TF motif enrichment
BinGO	3.0.3	Maere et al. (2005)	GO enrichment
Clustal Omega	1.2.4	Sievers et al. (2011)	Multiple sequence alignment
Cytoscape	3.3.0	Shannon et al. (2003)	Run BinGO
EdgeR	3.14.0	Robinson et al. (2010)	Analysing DEGs
Genevestigator		Hruz et al. (2008)	Public transcriptome data
Genesis	1.7.7	Sturn et al. (2002)	Heatmaps, clustering
Htseq	0.6.0	Anders et al. (2015)	Count RNA-seq reads
limma	3.28.14	Ritchie et al. (2015)	Analyzing DEGs
MixOmics	6.0	Rohart et al. (2017)	PCA
TopHat	2.1.1	Trapnell et al. (2009)	Map RNA-seq reads
VennDiagram	1.6.17	Chen and Boutros (2011)	Venn diagrams

The expression clusters of DEGs determined for the combined RNA-seq analysis of *A. thaliana* accessions together with Brassicaceae species were investigated for enrichment of GO terms corresponding to biological processes using the BinGO plugin within the Cytoscape environment. GO term enrichment was calculated using a hypergeometric test, followed by Benjamini and Hochberg FDR correction implemented in the BinGO plugin. The whole genome annotation was used as a background.

Known TF motifs enriched in individual expression clusters of DEGs determined in the combined RNA-seq analysis of *A. thaliana* accessions together with Brassicaceae species were identified using the AME tool within the MEME suite. For this purpose, 5′-gene regulatory regions (500 bp upstream of the transcription start site) were extracted for each tested Brassicaceae species. Enrichment of TF motifs was determined in each of the 15 *k*-means clusters for all tested Brassicaceae species using the 5′-regulatory regions of all expressed genes with clear 1:1 orthologs (16,100 genes) as a background. Known TF motifs were retrieved from the JASPAR CORE (2018) plants database that is implemented in AME.

To compare AA sequence conservation with expression variation, all AA sequences of expressed genes with 1:1 orthologs in all species were extracted for each Brassicaceae species. The sequences were aligned using Clustal Omega and percent identity matrices were extracted. The AA sequence identity output of Clustal Omega was used to calculate the mean AA identity across *C. rubella*, *C. hirsuta*, and *E. salsugineum* compared with *A. thaliana* as a proxy of sequence conservation. The mean AA sequence identities were subsequently plotted against the SD/mean of flg22-expression changes across all four Brassicaceae species, which served as a proxy for expression variation among the tested Brassicaceae species. Similarly, the mean AA sequence identity was also plotted against the SD/mean of the normalized expression value in control samples. In addition, pairwise AA sequence identities between *A. thaliana* and each Brassicaceae species were plotted against the absolute difference in flg22-induced expression changes between the compared species. This analysis was performed for all expressed genes or only for DEGs.

To calculate the dN, dS, and dN/dS ratios for DEGs, we aligned the AA sequences of each of orthogroup using MUSCLE with a maximum-likelihood approach (Edgar, 2004) and translated this alignment into the corresponding codon alignment with PAL2NAL (Suyama et al., 2006), also removing stop codons and gaps. The resulting codon alignments were used for pairwise dN, dS, and dN/dS rate calculations using the codeml tool from PAML (Yang, 2007). Software and packages used in this study are described in Table 6.

### Phylogenetic analysis

Various dates have been reported for the divergence of Brassicaceae species (Franzke et al., 2016). For instance, Beilstein et al. (2010) dated the Brassicaceae crown node age to 54 Mya, whereas more recent publications dated this event 31–37 Mya (Edger et al., 2015; Hohmann et al., 2015; Franzke et al., 2016; Huang et al., 2016). Therefore, in this study, we used TIMETREE (www.timetree.org), which synthesizes divergence times based on the available literature to estimate the timescale of Brassicaceae species evolution (Hedges et al., 2015). Phylogenetic trees were retrieved from timetree.org based on divergence time estimates from 15 studies (Koch et al., 2000; Heenan et al., 2002; Parkinson et al., 2005; Franzke et al., 2009; Yue et al., 2009; Beilstein et al., 2010; Couvreur et al., 2010; Mandáková et al., 2010; Arakaki et al., 2011; Hermant et al., 2012; Naumann et al., 2013; Artyukova et al., 2014; Vanneste et al., 2014; Hohmann et al., 2015; Huang et al., 2016).

### Genevestigator analysis

The following datasets were used for Genevestigator analysis: AT-00106 (*Pto* DC3000); AT-00110 (ABA or MeJA); AT-00113 (SA); AT-00147 (*B. cinerea*); AT-00253 (flg22 or OG); AT-00493 (hypoxia); AT-00553 (*Hyaloperonospora arabidopsidis*); AT-00560 (drought); AT-00597 (Pep2 and elf18); AT-00645 (heat stress).

### SA analysis

SA levels were analyzed as described previously with an ultra-high performance liquid chromatography/Q-Exactive system (Thermo Fisher Scientific) using an ODS column (AQUITY UPLC BEH C18, 1.7 μm, 2.1 × 100 mm; Waters, Milford, MA, USA) (Kojima et al., 2009; Kojima and Sakakibara, 2012; Yasuda et al., 2016).

### Secondary metabolite extraction, acquisition, and processing of data

Control and flg22-treated seedlings were collected and extracted as described before (Bednarek et al., 2011). The extracts were subjected to LC–MS analyses performed using the Acquity UPLC system (Waters) attached to a micrOToF-Q mass spectrometer (Bruker Daltonics, Hamburg, Germany). Chromatographic separations were carried out on a BEH C18 column (2.1 × 150 mm, 1.7 µm particle size) at 22°C with a mobile phase flow rate of 0.35 mL/min. The elution was conducted using water containing 0.1% formic acid (Sigma–Aldrich, Munich, Germany) (Solvent A) and acetonitrile (VWR Chemicals, Fontenay-sous-Bois, France) containing 1.9% of water and 0.1% of formic acid (Solvent B) in the following gradient: 0–10 min from 0% to 25% B, 10–15 min to 30% B, 20–24 min maintained at 100% B, and up to 24.5 min the system was returned to starting conditions and re-equilibrated for 8 min. The spectrometer was calibrated with sodium formate salt clusters prior to each analysis. MS was operated using the following settings: ion source voltage of −4.5or 4.5 kV, nebulization of nitrogen at a pressure of 1.2 bar and a gas flow rate of 8 L/min. Ion source temperature was 220°C. The spectra were scanned in positive and negative ion mode at a range of 50–1000 *m/z* at a resolution >15,000 FWHM (full width at half maximum). Data acquisition was supervised by HyStar version 3.2 software (Bruker Daltonics).

The LC–MS data were converted to *mzXML* format by MSConvert version: 3.0.11781 tool available in Proteowizard software prior to further processing by MZmine version 2.31 software (Pluskal et al., 2010). Data from each experiment were processed separately for negative and positive ionization. In first step, lists of masses were generated by the mass detector module in each scan in the raw data files. Chromatograms for each mass detected continuously over the scans were then built using a chromatogram builder algorithm. These chromatograms were deconvoluted by the deconvolution module using the wavelets algorithm based on Bioconductor’s XCMS package for R (Tautenhahn et al., 2008). An isotopic peaks grouper was used for isotope elimination followed by adduct and complex searching. Deviation of retention times between peak lists was reduced by a retention time normalizer. Such transformed peaks were aligned in all samples through a match score by a join aligner module. The resulting peaks list was completed by supplemental peak detection with a peak finder algorithm prior to missing value imputation (gap filling). The generated data table was subsequently exported in *csv* format for further statistical analysis.

Observations equal to zero (below the detection level) were substituted by half of the minimum non-zero observation for each metabolite. The observations were then transformed by log_2_(10^3^×). Two-way analysis of variance (ANOVA) was done with experiment as a block (random effects) and treatment, species as two fixed factors; analysis was done together for positive and negative ionization. A species was determined to be a DAM if it met all three conditions: (1) treatment effect or interaction treatment × species was significant with q-value <0.05 (FDR, Benjamini and Hochberg, 1995), (2) individual tests for each species *P* < 0.05 (significant test for the difference between treatment and control was done for each species), and (3) |fold change|>1.5, where fold change is flg22 treatment/control. Statistical analysis was performed in Genstat version 19. Visualizations including barplot, PCA, heatmap, and Venn diagram were created in R.

### Intra specific variation

Analyses of intra specific variation in *A. thaliana* were conducted using a subset of the accessions from the 1001 Genome Project (1001 Genomes Consortium, 2016). To minimize the potentially confounding effects of demography and admixture, we only selected accessions from the non-relict Iberian ancestry group with ˂5% admixture from other groups (http://1001genomes.github.io/admixture-map/), which led to a sample of 45 accessions. Genomic regions for genes and upstream regions were identified using the REST API of the Ensembl database (https://rest.ensembl.org/). For each gene, the longest available transcript was used. Genetic divergence between *A. thaliana* and *A. lyrata* was calculated using the genome-wide pairwise alignment available on Ensembl Plant (LastZ) and also accessed through their REST API server. The code used for interacting with the REST APIs and calculating summary statistics of polymorphism and divergence data is written in R for this study and is made publicly available here: https://gitlab.mpcdf.mpg.de/slaurent/mk_dfe.git.

To determine whether mean genetic divergence between *A. thaliana* and *A. lyrata* was significant (Supplemental Figure S10) across clusters, we performed a one-way ANOVA with gene expression clustering as the grouping factor. We used the *anova_test* function from the *rstatix* package; the R code implementing this function is publicly available in the following repository: https://gitlab.mpcdf.mpg.de/slaurent/mk_dfe.git in the folder *anova_figureS9.*

### Hierarchical clustering

RNA-seq mapped read counts of 1:1 orthologs were subjected to TMM normalization after removing low-expression orthologs (average count of <10 reads). A pairwise distance matrix (1 – Pearson’s correlation coefficient) was obtained from log_2_ fold changes of CPM values. A dendrogram was generated with the R package “pvclust” with default settings. The same analysis was performed using log_2_ fold changes of DAMs for metabolomic comparison.

### OU modeling

OU modeling was performed using the R package “l1ou” (Khabbazian et al., 2016) with the following parameters: max.nShifts = 1, criterion = pBIC, root.model = OUfixedRoot, rescale = FALSE, and alpha.upper = 0.053. The upper limit of *α* estimates was calculated by the find_grid_alpha function in the R package “PhylogeneticEM” (Bastide et al., 2018).

## Accession numbers

The RNA-seq data used in this study were deposited in the National Center for Biotechnology Information Gene Expression Omnibus database (accession no. GSE115991).

## Supplemental data

The following materials are available in the online version of this article.

**Supplemental Figure S1** Effects of flg22 on *Pto hrcC* growth in Brassicaceae species.

**Supplemental Figure S2** Overlap of DEGs at each time point.

**Supplemental Figure S3** *SID2*-dependent SA accumulation is not required for sustained transcriptome responses in *A. thaliana*.

**Supplemental Figure S4** Heatmap for all DEGs in Brassicaceae species after flg22 treatment.

**Supplemental Figure S5** Comparison of two different mapping approaches for *A. thaliana* accession RNA-seq reads.

**Supplemental Figure S6** Gene family size and basal gene expression levels do not explain species-specific expression signatures.

**Supplemental Figure S7** Multi-optima phylogenetic OU modeling of log_2_ fold changes of 1:1 orthologs in Figure 5, A.

**Supplemental Figure S8** Enrichment of TF-motifs within the 5′-regulatory regions of DEG clusters.

**Supplemental Figure S9** Gene expression variation does not correlate with dN/dS variation.

**Supplemental Figure S10** Genetic divergence between *A. thaliana* and *A. lyrata* for upstream, synonymous, and non-synonymous sites.

**Supplemental Data Set S1.** RNA-seq read counts for 1:1 orthologs in all the samples used in this study.

**Supplemental Data Set S2.** Expression changes (log_2_) of DEGs in Figure 3, A.

**Supplemental Data Set S3.** Expression changes (log_2_) of DEGs in Figure 3, C.

**Supplemental Data Set S4.** GO analysis of DEGs in Figure 3, D.

**Supplemental Data Set S5.** Expression changes (log_2_) of DEGs in Figure 4, G.

**Supplemental Data Set S6.** Expression changes (log_2_) of DEGs in Figure 5, A.

**Supplemental Data Set S7.** Enrichment analysis of known TF-binding motifs in the 5′-regulatory regions of genes in shared and species-specific clusters.

**Supplemental Data Set S8.** GO analysis of gene clusters in Figure 5, A.

**Supplemental Data Set S9.** Primers used in this study.

## Supplementary Material

koab073_Supplementary_Data

## Notes

T.M.W., S.A., P.S.L., P.B., and K.T. designed the research. T.M.W., F.E., S.A., A.P., and K.K. performed the research. H.S., X.G., M.T., and R.G.-O. contributed new reagent/analytic tools. T.M.W., S.A., F.E., A.P., B.S., E.D., A.S., K.F., S.L., P.B., and K.T. analyzed the data. T.M.W., F.E., and K.T. wrote the paper with input from all authors.

The author responsible for the distribution of materials integral to the findings presented in this article in accordance with the policy described in the Instructions for Authors (https://academic.oup.com/plcell) is Kenichi Tsuda (tsuda@mail.hzau.edu.cn).
