# Supplementary material for: Gene expression evolution in pattern-triggered immunity within Arabidopsis thaliana and across Brassicaceae species
Source: Plant Cell. 2021 Mar 5;33(6):1863–87. doi: 10.1093/plcell/koab073 (PMC8290292; doi:10.1093/plcell/koab073)
Supplement: koab073_Supplementary_Data [file koab073_supplementary_data.zip › tpc.00600.2020-s01.pdf]

Supplemental Data. Winkelmüller, Entila, Anver et al. (2021). Gene expression evolution in pattern-triggered immunity within *Arabidopsis thaliana* and across Brassicaceae species. Plant Cell.

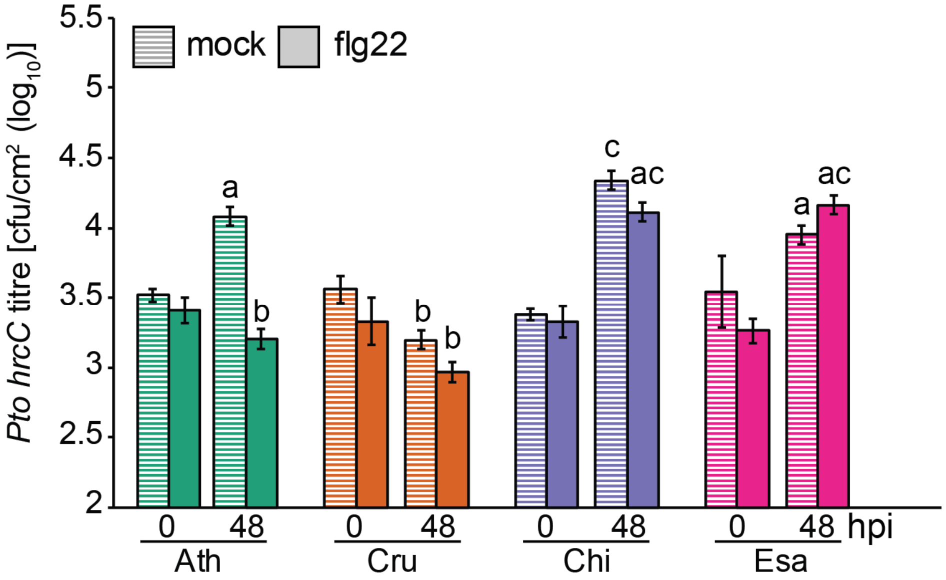

Supplemental Figure 1. Effects of flg22 on *Pto hrcC* growth in Brassicaceae species. (Supports Figure 1)
Leaves of 5-week-old plants were syringe-infiltrated with mock or 1 µM flg22 24 h prior to infiltration with *Pto hrcC* (OD_{600} = 0.001). The bacterial titre was determined 0 and 48 h after bacterial infiltration. Bars represent means and SEs from 2 independent experiments each with 12 biological replicates (n = 24). Different letters indicate statistically significant differences (mixed linear model, adjusted $p < 0.01$). Ath, *A. thaliana* (Col-0); Cru, *C. rubella* (N22697); Chi, *C. hirsuta* (Oxford); Esa, *E. salsugineum* (Shandong).

Supplemental Data. Winkelmüller, Entila, Anver et al. (2021). Gene expression evolution in pattern-triggered immunity within *Arabidopsis thaliana* and across Brassicaceae species. Plant Cell.

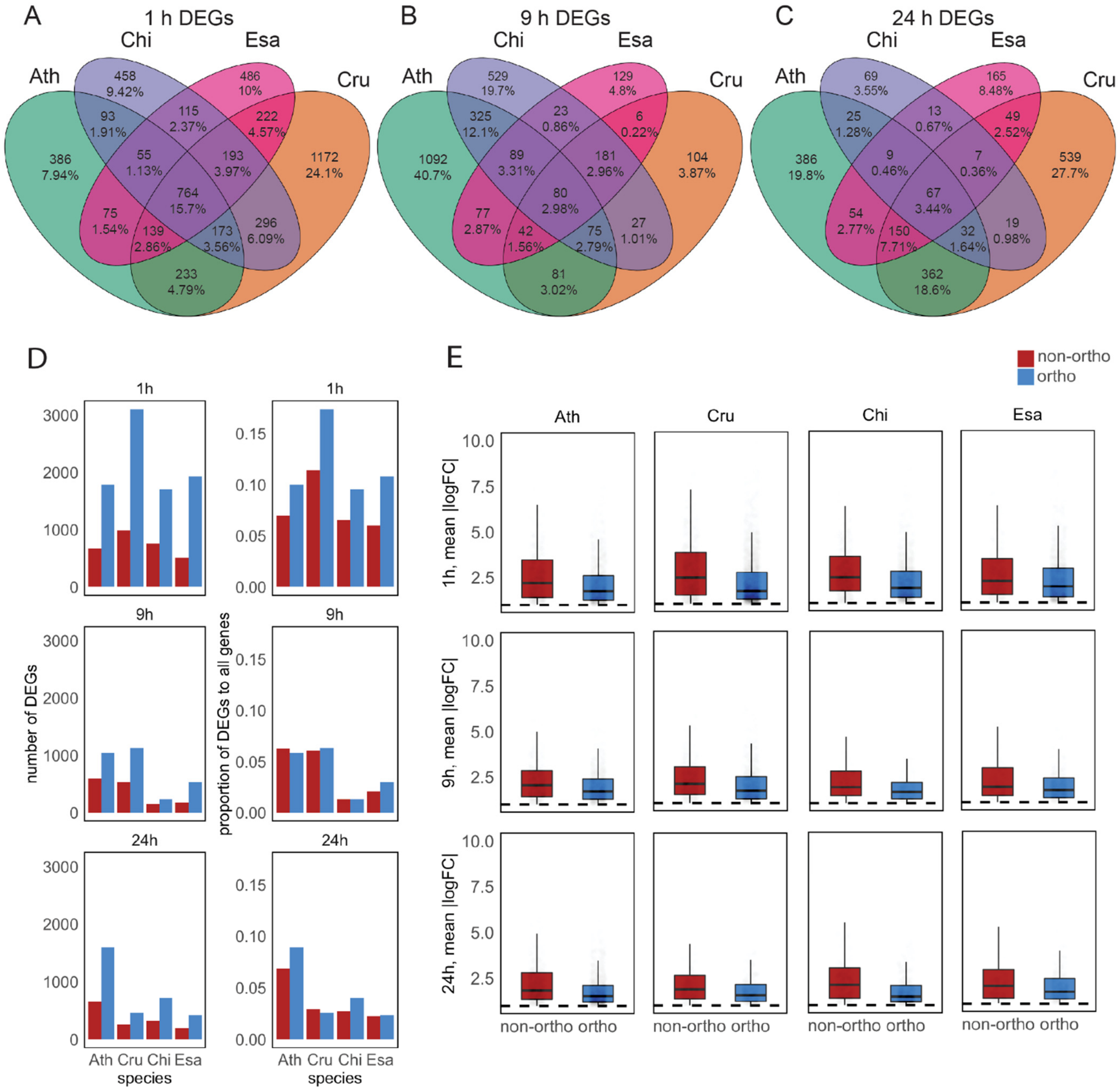

Supplemental Figure 2. Overlap of DEGs at each time point. (Supports Figure 2) Venn diagrams showing shared and specific DEGs between species at 1 h (**A**), 9 h (**B**) and 24 h (**C**) after flg22-treatment. All DEGs differentially expressed in at least species at the respective time points were used. Ath, *A. thaliana* (Col-0); Cru, *C. rubella* (N22697); Chi, *C. hirsuta* (Oxford); Esa, *E. salsugineum* (Shandong). (**D**) The comparison of the number and the proportion of DEGs relative to all genes (including both DEGs and non-DEGs) for genes with or without 1:1 orthologue assignment for each time point. (**E**) The mean absolute log_2-fold change (|logFC|) of DEGs for genes with and without 1:1 orthologue assignment for each time point.

Supplemental Data. Winkelmüller, Entila, Anver et al. (2021). Gene expression evolution in pattern-triggered immunity within *Arabidopsis thaliana* and across Brassicaceae species. Plant Cell.

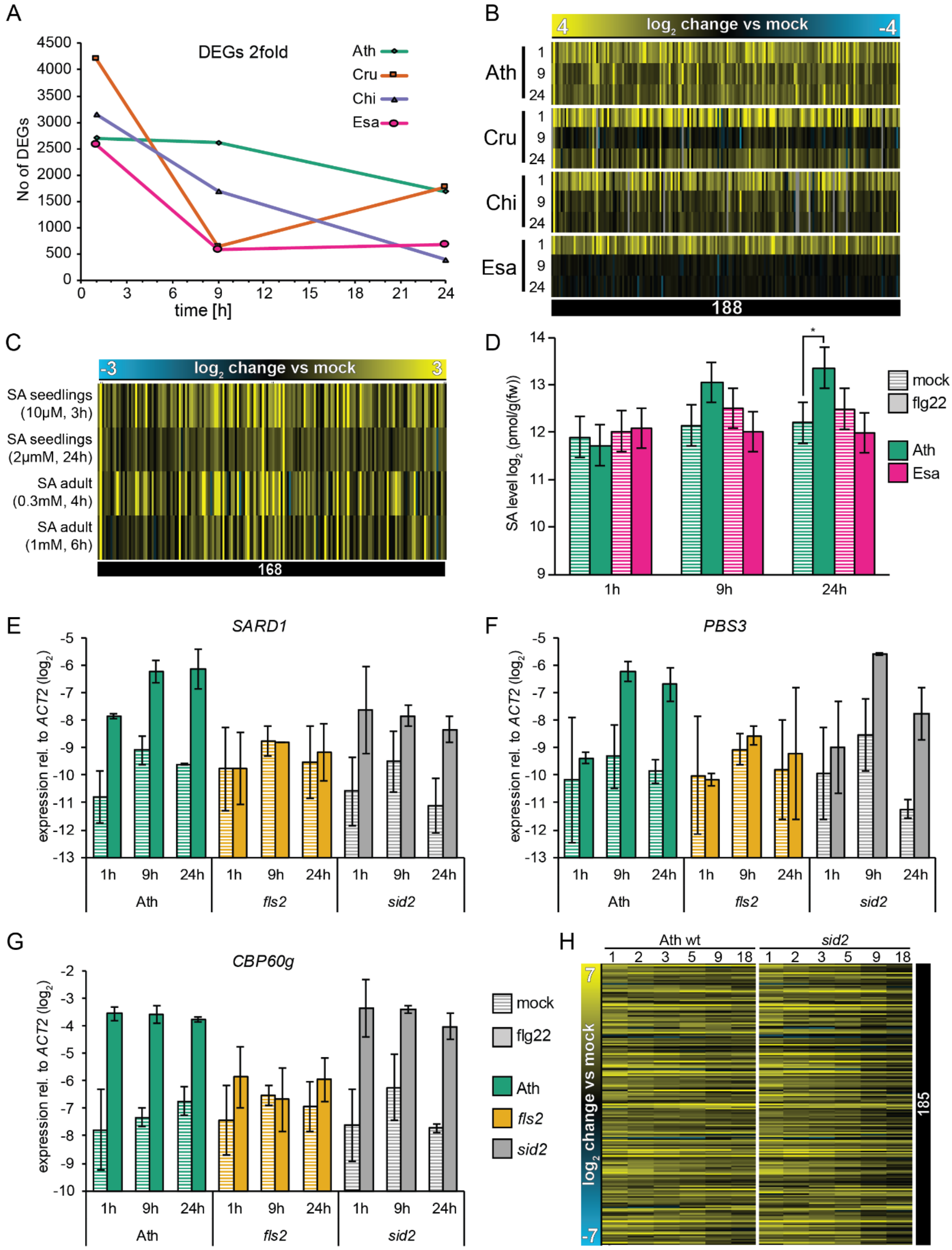

Supplemental Data. Winkelmüller, Entila, Anver et al. (2021). Gene expression evolution in pattern-triggered immunity within *Arabidopsis thaliana* and across Brassicaceae species. Plant Cell.

Supplemental Figure 3. *SID2*-dependent SA accumulation is not required for sustained transcriptome responses in *A. thaliana*. (Supports Figure 3) (**A**) The temporal dynamics of the transcriptional response to flg22 differs in Brassicaceae species. The numbers of DEGs (q-value < 0.01; |log2 fold change| > 1) at each time point in each species are plotted. (**B**) Heatmap visualizing 188 genes induced at 1 hpt in Ath and Esa (log2 induction > 0.6) with sustained induction in Ath (log2 induction > 0.6 at 9 and 24 hpt) but transient induction in Esa (log2 induction < 0.5 at 9 and 24 hpt). (**C**) Most of the 188 genes (missing genes are due to missing probes on microarrays of public datasets) are responsive to SA in publicly available expression data of *A. thaliana* (Genevestigator). (**D**) Free salicylic acid (SA) levels of 12-day-old seedlings were determined using HPLC-MS at the indicated time-points after mock or 1 µM flg22 treatment. Bars represent the means ±SE from 3 independent experiments. Asterisks indicate significant difference to mock (mixed linear model followed by Student's t-test; *, p < 0.05). (**E – G**) 12-day-old seedlings of *A. thaliana* Col-0 wild type, *fls2*, and *sid2* were treated with mock or 1 µM flg22 for 1, 9, or 24 h. Expression of 3 marker genes extracted from the heatmap in Figure 4B, namely *SARD1* (**E**), *PBS3* (**F**) and *CBP60g* (**G**) was quantified using RT-qPCR. Bars represent the means ±SD from 2 independent experiments. Ath, *A. thaliana* (Col-0); Cru, *C. rubella* (N22697); Chi, *C. hirsuta* (Oxford); Esa, *E. salsugineum* (Shandong). (**H**) The 188 genes in Figure 4B showing transient induction in Esa were analyzed for their expression induction in 31 to 32-day-old Col-0 and *sid2* leaves at the indicated time points compared to 0 h after 1 µM flg22 treatment (Hillmer et al., 2017).

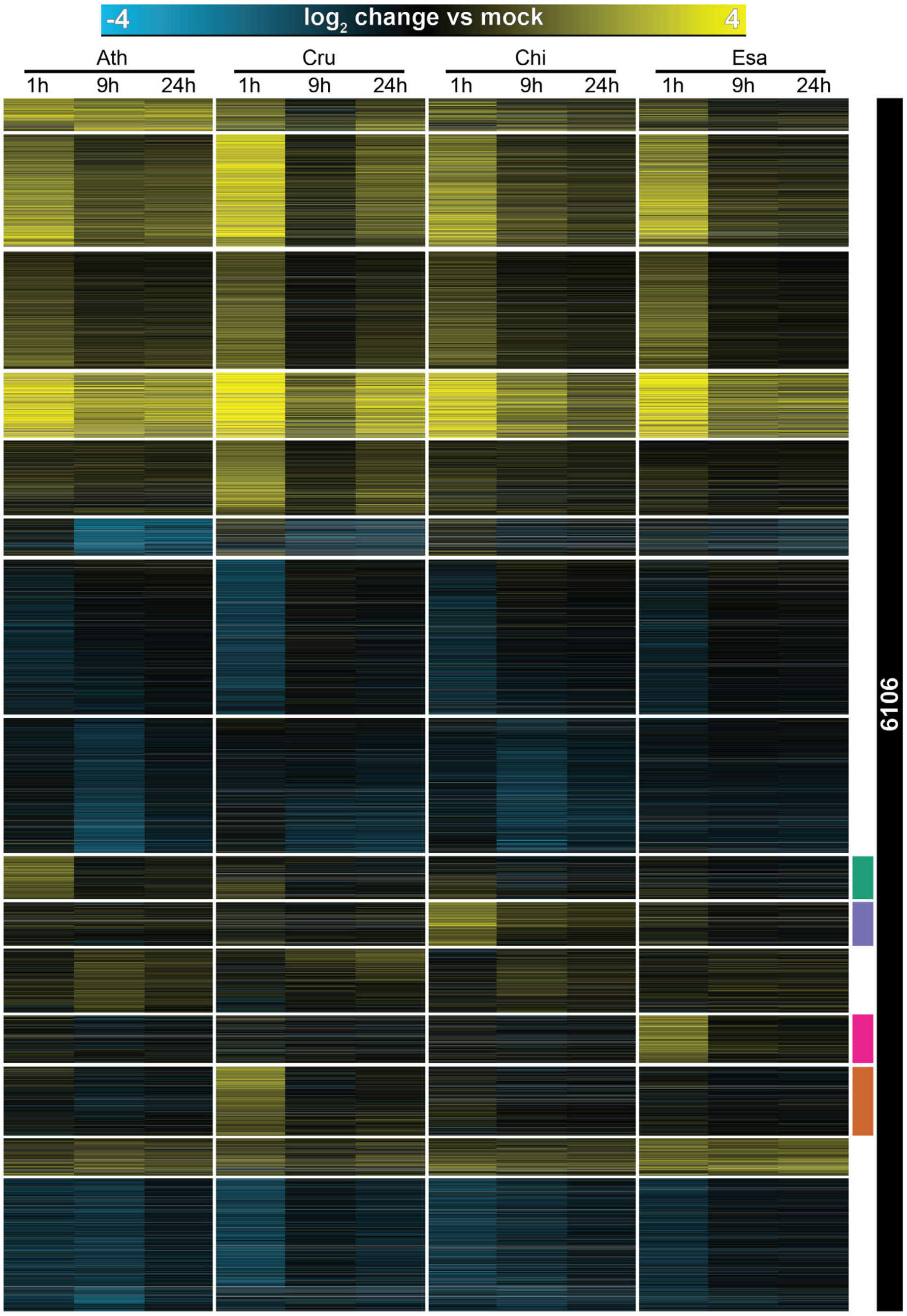

Supplemental Figure 4. Heatmap for all DEGs in Brassicaceae species after flg22 treatment. (Supports Figure 3) Heatmap of $\log_2$ fold changes for all 6,106 DEGs among all Brassicaceae species generated using k-means clustering (k = 15). All DEGs which are differentially expressed at least at 1 time point in 1 species were used. Species-specific expression clusters shown in Figure 3C are indicated by coloured bars [Ath (green), Cru (orange), Chi (purple), and Esa (magenta)] on the right side. Ath, *A. thaliana* (Col-0); Cru, *C. rubella* (N22697); Chi, *C. hirsuta* (Oxford); Esa, *E. salsugineum* (Shandong).

Supplemental Data. Winkelmüller, Entila, Anver et al. (2021). Gene expression evolution in pattern-triggered immunity within *Arabidopsis thaliana* and across Brassicaceae species. Plant Cell.

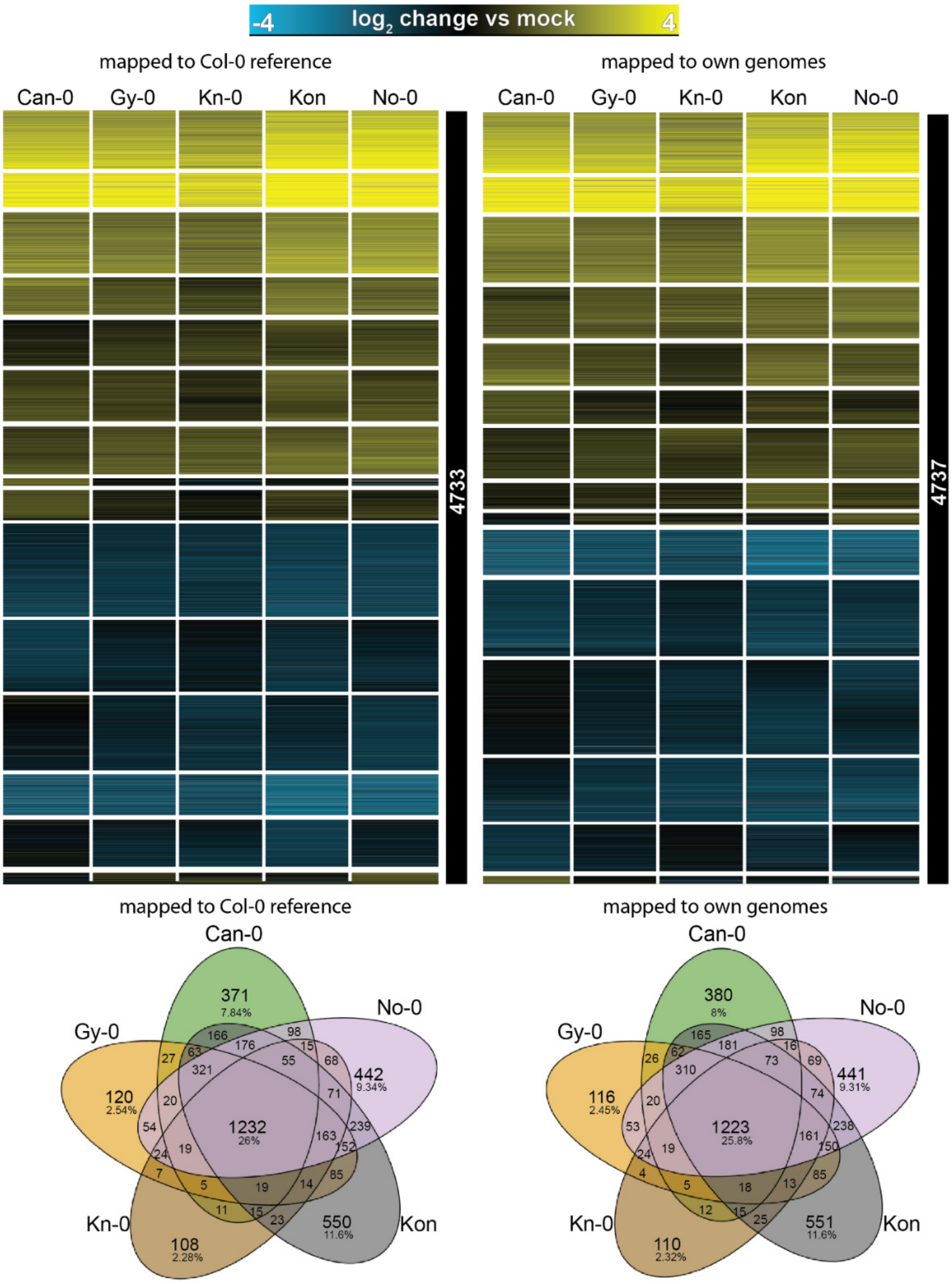

Supplemental Figure 5. Comparison of two different mapping approaches for *A. thaliana* accession RNA-seq reads. (Supports Figure 4) RNA-seq reads were mapped to the Col-0 (TAIR10) reference genome (left) or to individual *A. thaliana* accession genomes generated in this study using SNP data (right). Heatmap of DEGs in at least 1 accession clustered by k-means (k = 15). The log_2 expression changes compared to mock are shown.

Supplemental Data. Winkelmüller, Entila, Anver et al. (2021). Gene expression evolution in pattern-triggered immunity within *Arabidopsis thaliana* and across Brassicaceae species. Plant Cell.

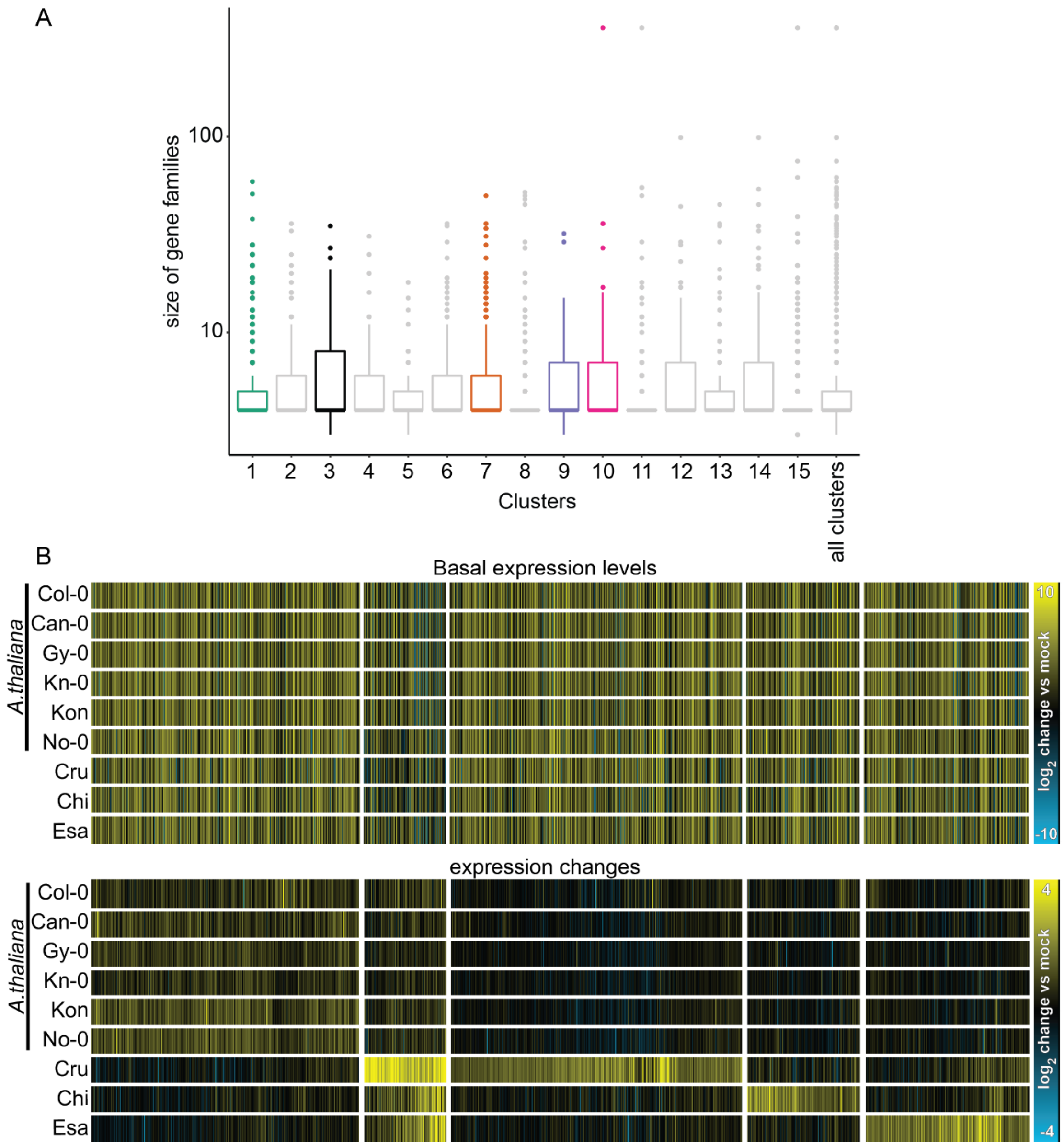

Supplemental Figure 6. Gene family size and basal gene expression levels do not explain species-specific expression signatures. (Supports Figure 5) (**A**) The sum of the number of genes in gene families among the 4 tested Brassicaceae species are plotted at log_{10} scale for each of the 15 clusters obtained by k-means clustering of all 5,961 genes that are DEGs in at least one species or accession (See Figure 5). Species-specific clusters are highlighted by colours [Ath (green), Cru (orange), Chi (purple) and Esa (magenta)]. Cru, *C. rubella* (N22697); Chi, *C. hirsuta* (Oxford); Esa, *E. salsugineum* (Shandong)]. (**B**) Basal (mock condition) expression levels (normalized and log_2-transformed counts per million) of genes showing species-specific expression signatures are shown in the upper heatmap. The log_2 expression changes after flg22 treatment are shown in the bottom heatmap.

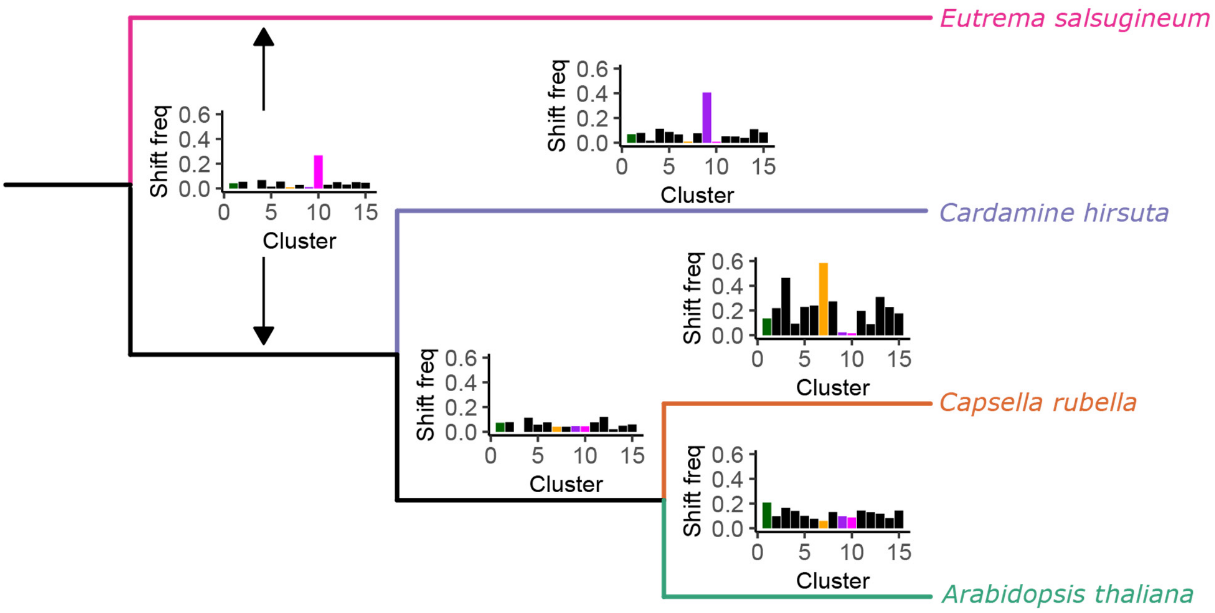

Supplemental Figure 7. Multi-optima phylogenetic Ornstein-Uhlenbeck (OU) modelling of log_2 fold changes of 1:1 orthologues in Figure 5A (supports Figure 5). The frequencies of detected regime shifts in each cluster (Figure 5A) are shown in bar plots on branches. Colours of the bars represent the species-specific clusters similar to Figure 5 (*Ath*- green, *Cru*- orange, *Chi*- purple, and *Esa*- magenta). It is impractical to technically separate the shifts in the sub-root branches (i.e., the branch connected to *E. salsugineum* and the stem branch connected to the other three species), and therefore those branches were not distinguished in this analysis (indicated by arrows).

Supplemental Data. Winkelmüller, Entila, Anver et al. (2021). Gene expression evolution in pattern-triggered immunity within *Arabidopsis thaliana* and across Brassicaceae species. Plant Cell.

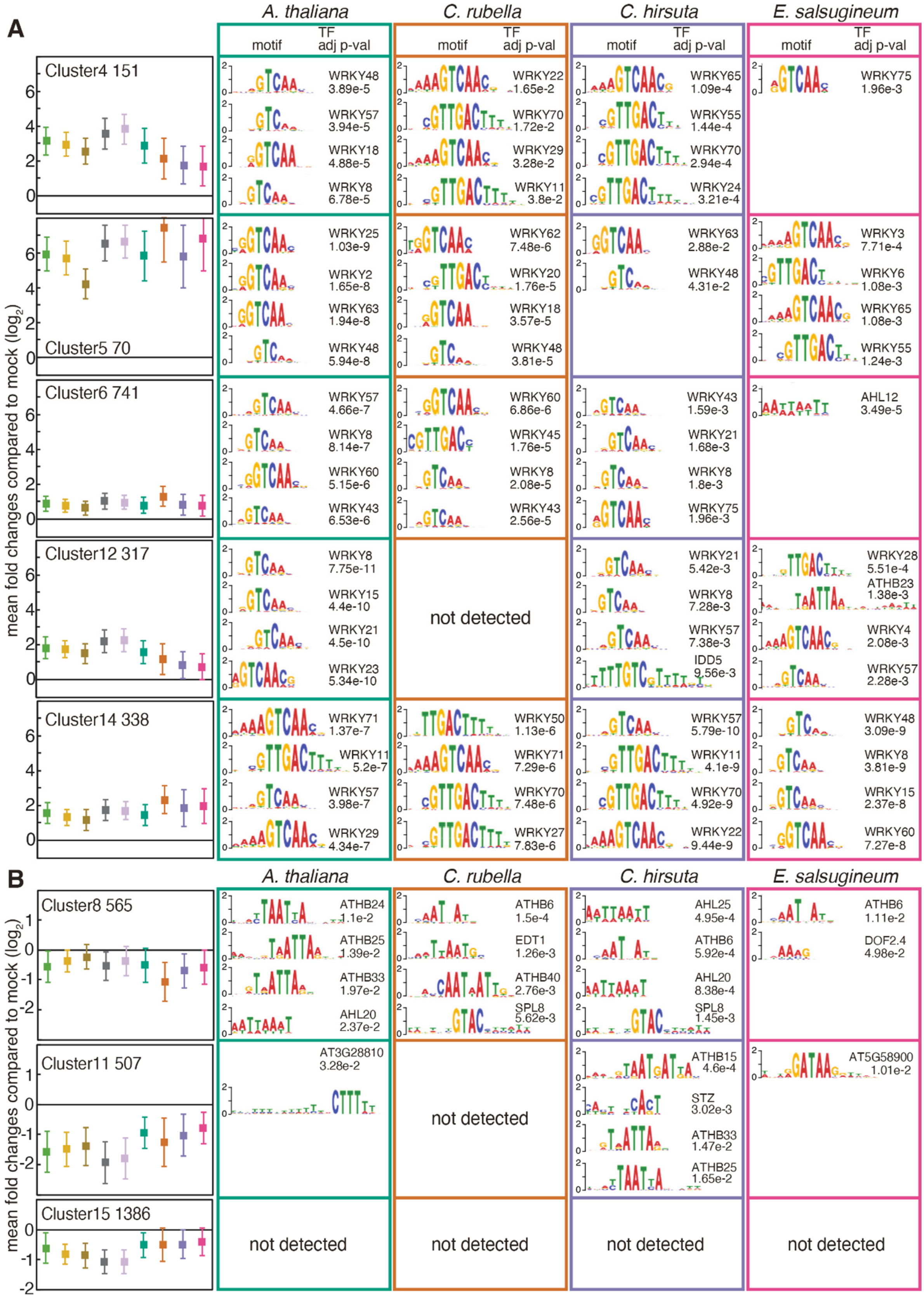

Supplemental Figure 8. Enrichment of TF-motifs within the 5′-regulatory regions of DEG clusters. (Supports Figure 7) The 500 bp upstream sequences of the transcription start sites of the genes in the individual clusters were tested for enrichment of known TF binding motifs. Names of transcription factors, sequence logos and adjusted p-values (up to the top 4) of motifs are shown for each Brassicaceae species. The names of clusters, the number of DEGs, and mean $\log_2$ fold changes ±SD compared to mock are shown on the left side. For the complete list of all enriched TF binding motifs, please see Supplemental Data Set 6. Ath, *A. thaliana* (Col-0); Cru, *C. rubella* (N22697); Chi, *C. hirsuta* (Oxford); Esa, *E. salsugineum* (Shandong). A: Commonly induced clusters. B: Commonly downregulated clusters.

Supplemental Data. Winkelmüller, Entila, Anver et al. (2021). Gene expression evolution in pattern-triggered immunity within *Arabidopsis thaliana* and across Brassicaceae species. Plant Cell.

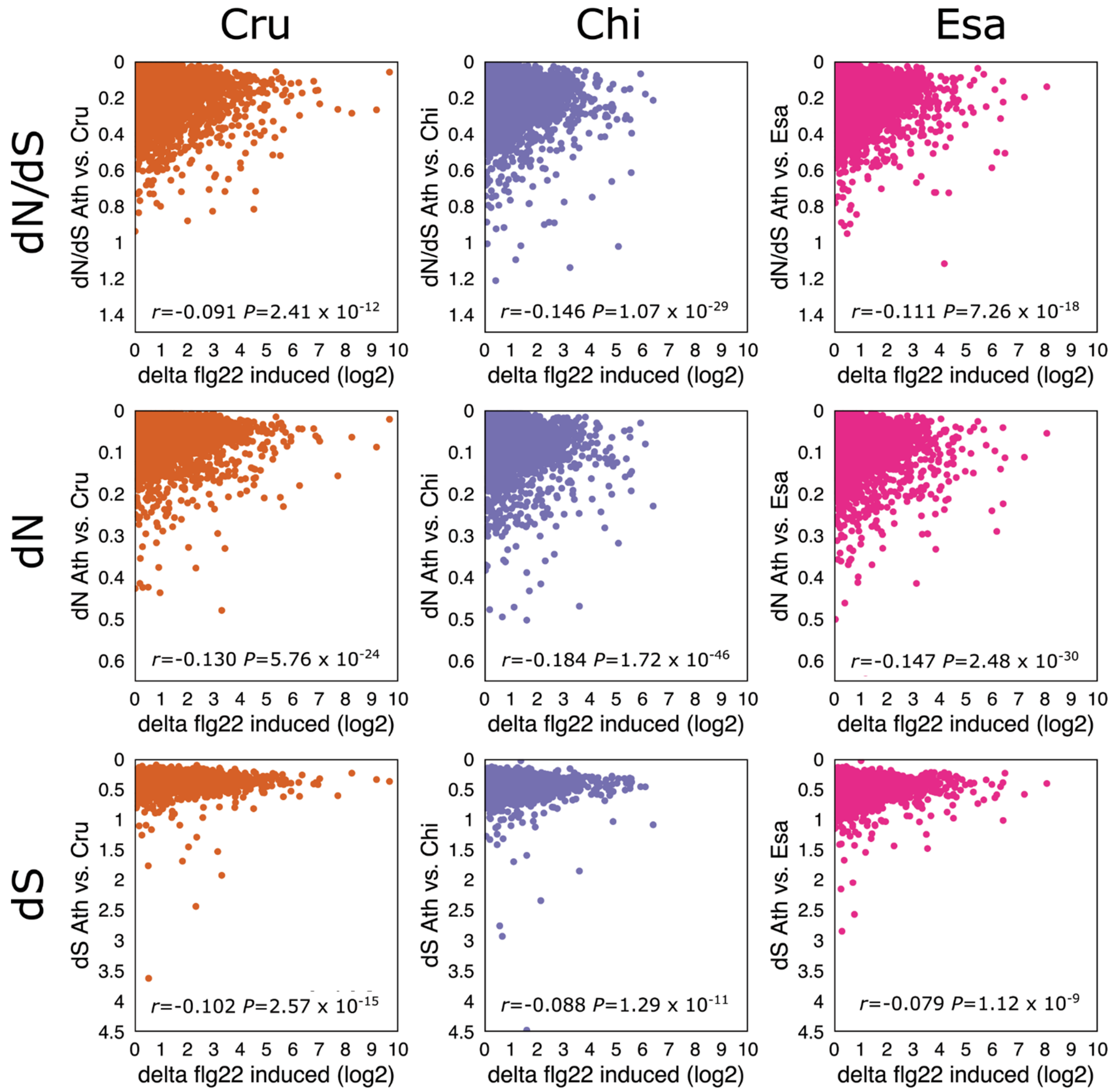

Supplemental Figure 9. Gene expression variation does not correlate with dN/dS variation. (Supports Figure 8) dN, dS and dN/dS of *C. rubella* (Cru), *C. hirsuta* (Chi) and *E. salsugineum* (Esa) to *A. thaliana* (Ath) were plotted against the flg22-induced expression changes between the compared species for 5,961 DEGs.

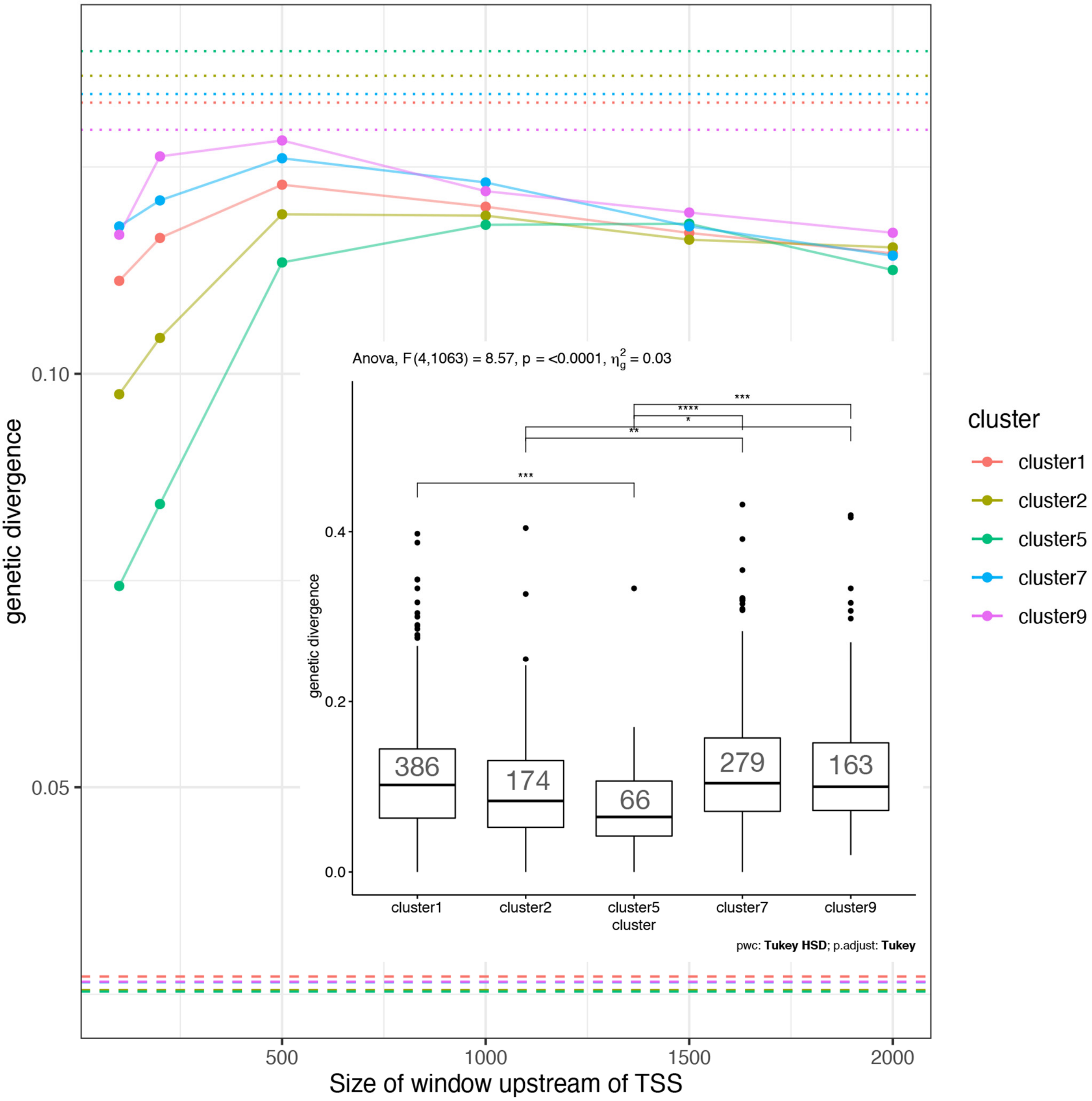

Supplemental Figure 10. **Genetic divergence between *A. thaliana* and *A. lyrata* for upstream, synonymous, and non-synonymous sites. (Supports Figure 8)** The y-axis shows the proportion of sites that are different between the reference sequences of *A. thaliana* and *A. lyrata*. The x-axis indicates the size of the window upstream of the SFS used to calculate the "upstream" genetic divergence (solid lines). The dashed and dotted lines represent the non-synonymous and synonymous genetic divergences, respectively. The inset shows the result of a one-way ANOVA testing differences in mean divergence between the five clusters when windows of 100 bp are considered. Numbers in the boxplots represent the number of 100 bp-windows present in each distribution.
