# Supplementary material for: Gene expression evolution in pattern-triggered immunity within Arabidopsis thaliana and across Brassicaceae species
Source: Plant Cell. 2021 Mar 5;33(6):1863–87. doi: 10.1093/plcell/koab073 (PMC8290292; doi:10.1093/plcell/koab073)
Supplement: koab073_Supplementary_Data [file koab073_supplementary_data.zip › tpc.00600.2020-s12.pdf]

# Gene expression evolution in pattern-triggered immunity within *Arabidopsis thaliana* and across Brassicaceae species

Thomas M. Winkelmüller, Frederickson Entila, Shajahan Anver, Anna Piasecka, Baoxing Song, Eik Dahms, Hitoshi Sakakibara, Xiangchao Gan, Karolina Kułak, Aneta Sawikowska, Paweł Krajewski, Miltos Tsiantis, Ruben Garrido-Oter, Kenji Fukushima, Paul Schulze-Lefert, Stefan Laurent, Paweł Bednarek, and Kenichi Tsuda

*Corresponding author: Kenichi Tsuda tsuda@mail.hzau.edu.cn*

**Review timeline:**

| | | |
|---|---|---|
| **TPC2020-LSB-00600** | Submission received: | July 29, 2020 |
| | 1st Decision: | Sept. 3, 2020 *revision requested* |
| **TPC2020-LSB-00600R1** | 1st Revision received: | Oct. 30, 2020 |
| | 2nd Decision: | Dec. 14, 2020 *revision requested* |
| **TPC2020-LSB-00600R2** | 2nd Revision received: | Jan. 15, 2021 |
| | 3rd Decision: | Feb. 8, 2021 *accept with minor revision* |
| **TPC2020-LSB-00600R3** | 3rd Revision received: | Feb. 10, 2021 |
| | 4th Decision: | Feb. 10, 2021 *acceptance pending, sent to science editor* |
| | Final acceptance: | Feb. 24, 2021 |


**REPORT:** (The report shows the major requests for revision and author responses. Minor comments for revision and miscellaneous correspondence are not included. The original format may not be reflected in this compilation, but the reviewer comments and author responses are not edited, except to correct minor typographical or spelling errors that could be a source of ambiguity.)

**TPC2020-LSB-00600 1st Editorial decision – *revision requested* Sept. 3, 2020**

The editors and reviewers agreed that a number of the evolutionary claims are not supported by an evolutionary analysis. For example, the PCA to expected phylogeny was not considered as the proper test. It is possible to utilize the data in a quantitative way to conduct phylogeny with the transcript traits directly, as pointed out be a reviewer. The phylogeny to PCA would need to be corrected with analysis using the existing data.

Additionally, claims of purifying or other selection have specific quantitative comparison to the whole genome that were not conducted. After discussion amongst the editors and reviewers, it was agreed that the claims on selection would require extensive work to properly test and that the novelty of the manuscript does not rely on these claims. As such, it possible to remove them. Alternatively, if you would like to retain them, they will require empirical testing with new analysis for support.

Finally, there was a general concern about only focusing on one-to-one orthologues and how this may influence the claims. And, related to this, is a desire for more discussion on the difficulties of mapping across even closely related species and how this may influence the analysis. In general, the editors and reviewers felt that making the claims on purely the one-to-one orthologues means that there could be a significant bias in these terms. It would be possible to go into the non-one-to-one groups and simply ask if the fraction of responsive transcripts is higher or similar to the one-to-one group. We ask that you consider this also in your response.

--------------------------------------------------------------------------- Reviewer comments:
Reviewer #1 (Comments for the Author):

This manuscript sets out to investigate the genetic basis of pattern-triggered immunity in Brassicaceae species and link patterns of differential gene expression to selective processes. They use transcriptomes from 6 *Arabidopsis*

*thaliana* accessions and three other Brassicaceae species (*Capsella rubella*, *Cardamine hirsuta*, and *Eutrema salsugineum*) to identify genes with differential expression in response to flg-22, a microbial associated protein at three time points after treatment. They argue that expression changes are under purifying selection in Brassicaceae but that there is also evidence for directional selection on response. Additionally, they find transcription factor binding site enrichments in genes with flg-22 response.

This manuscript tackles an interesting topic of understanding how selection shapes pathogen responses, integrating a wide range of techniques from plant pathology to population genetics. I think that studies like this have the potential to tell us a lot about the adaptation of pathogen responses. However, the broad conclusion that purifying selection and directional selection shape the transcriptome responses to pathogens are not well supported. I describe why in detail below. In addition, I describe a few methodological concerns, as well as some concerns about limited information in the methods section.

1. I have a number of concerns about the inference of selection made in this paper. First, the abstract argues that there is a set of genes with expression responses under purifying selection in Brassicaceae. However, the main result for this claim seems to be that the 868 genes that show differential expression in response to flg-22 in the species studied. However, without any model for the amount of conservation of expression, it's not clear to me why 868 is more than expected under neutrality. To make this claim, the authors need to provide evidence that the number of genes with conserved expression responses is more than expected under drift. Otherwise, I think the strongest statement would be that conservation of the response is "consistent with neutral evolution or stabilizing selection" (I generally hear 'purifying selection' used to describe selection against new mutations, so this means selection on specific genetic variants. I think for a trait like gene expression, it would be more accurate to say "stabilizing selection" constrains trait evolution. But I realize that these terms are used in different ways.)

2. Second, the abstract claims that many genes show species-specific expression signatures. The evidence for this claim comes from an analysis comparing phylogenetic distances to a PCA of differentially expressed genes. The manuscript argues that because the PCA does not match phylogenetic differences there must have been directional selection (Fig 5e). However, I disagree that PCA of the expression of all differentially-expressed-in-flg-22 genes is the correct method to use here since it will be dominated by the genes with the most expression variation between species, potentially contributing to a distorted view of how much evolution there has been. This conclusion would be convincing if the manuscript used phylogenetic methods to test for selection on specific branches of the phylogeny (for an example see Nourmohammad et al. 2017 https://doi.org/10.1016/j.celrep.2017.07.033 and citations within). I also think that looking gene-by-gene would be a better approach than using a PCA.

3. The manuscript claims that there is stronger purifying selection acting on the upstream regions of genes in cluster 5 (highly induced in all species) than for other clusters (paragraph starting line 426). However, this result is not very convincing without a statistical comparison at least showing that the clusters differ from each other. I'm also curious why the authors chose *thaliana-lyrata* divergence here instead of divergence between more distantly related species, since it may be that *thaliana* and *lyrata* are too recently diverged to see large differences in constraint between genes.

4. In the "Variation in coding sequences show no strong correlation with transcriptome variation" section, the authors investigate whether sequence divergence correlates with expression level. They use total sequence similarity to measure sequence conservation, but much of the literature (including cited paper Hunt et al.) are looking specifically at dN and dN/dS. Could this explain why the authors here do not see a relationship? I'd also encourage the authors to cite more of the plant literature on this topic (for example Slotte et al. 2011 https://doi.org/10.1093/gbe/evr094, Hodgins et al 2016 https://doi.org/10.1093/molbev/msw032 and others). I'd also note that the available evidence suggests that the relationship between sequence divergence and expression seems to be due to stronger negative selection on more highly selected genes, so it's also perhaps unsurprising to not see this relationship in expression that is induced in specific environments.

5. This manuscript treats differential expression as binary -- either a gene is differentially expressed (adjusted p < 0.01 and minimum fold change is 2) or it isn't differentially expressed. However, I wonder if this choice means that the authors are missing out on shared transcriptome responses where in one species the response is just slightly below their cutoff. One potential way around this would be to see how robust the results are to changes in the cutoff used to measure differential expression. Alternatively, the authors could investigate the correlation in responses between species for all genes, not just those meeting their significance cutoff.

6. I am curious about what is being lost by only looking at genes with orthologs across all species. I understand that it's harder to compare these, but it would be useful to at least know what percentage of differentially expressed genes are not being included because they lack orthologs.

7. The manuscript reports that genes with shared differential expression across species were enriched for relevant GO terms, but that genes with species-specific responses did not show many GO enrichments (lines 244-248). Could this just be the result of the fact that GO terms are based on function as found in Arabidopsis so, if a gene is important for defense response in a non-Arabidopsis species only, it won't be annotated? I also find it confusing that this paragraph reports GO terms for the species-specific cluster even though there is not a significant enrichment.

8. The methods section is missing key information. I couldn't find information about the sample size for the differential expression experiments. I also couldn't find any information in the methods for the section starting on line 424. It is essential to know how they measured divergence between *A. thaliana* and *A. lyrata,* along with how Ka/Ks and pi_a/pi_s were calculated from the 1001 genome data.

Reviewer #2 (Comments for the Author):

This manuscript analyzes and characterizes a large dataset of gene expression profiles of Brassicaceae species after PAMP trigger. They identify both common and distinct gene expression patterns as well as shared transcription factor binding sites associated with these genes. How gene expression during pathogen infection is regulated is of broad interest to the plant pathogen community and the results should be made available to the community. However, while the observations made in this manuscript are of broad interest, they do not support many of the conclusions drawn from them. The authors over-conclude at many points, presenting their hypotheses as facts. While I think the data of the manuscript is valuable and should be published, it almost seems that the authors aren't overly familiar with evolutionary studies and tend to reach conclusions that aren't supported by data in this manuscript. I would therefore suggest the authors rewrite the manuscript and drastically soften their conclusions in order to publish it as is.

In the manuscript, the authors dissect the MAMP responses of four different Brassica species.
They did transcriptomic analyses to identify differentially expressed genes (DEGs) at 1h, 8h and 24h post flg22 treatment. The genes were clustered into groups of genes that were either differentially expressed in all four species or in a species-specific manner. While a large number of these commonly differentially expressed genes are known flg22-responsive genes with an established role in immunity, around 50% of these genes either do not have an established role in immunity or are unannotated. This study therefore has the potential to provide a large set of genes with a possible important role in immunity. However, since quite many of the genes that show differential expression in a species-specific manner are associated with processes distinct from plant immunity, the number of new plant immunity genes could be substantially lower than the total number of species-specific DEGs identified in this study. They also analyzed the gene expression changes in several geographically diverse Arabidopsis accessions. In addition to the gene expression variation, the authors also looked at genetic variation upstream of coding regions and transcription factor binding sites. However, none of these analyses yielded any interesting observations, so they could be replaced. Much more interesting is the metabolome profiles of the Brassicaceae. This is a very interesting dataset and can be expanded on more. I think this would be of very great interest to the community and can replace some of the poorer evolutionary analyses.

Major comments:
- The authors suggest that the regulation of the common set of differentially expressed genes is under purifying selection. However, they later analyse the regulatory regions of these gene clusters and don't find differences in genetic variation between clusters of DEGs. This seems to indicate that the mechanisms driving differential gene expression can't be explained by evolutionary pressure. Overall, the findings don't necessarily support the conclusions drawn on evolutionary mechanisms and pressures. I would suggest the authors rewrite the manuscript and soften some of their conclusions.
- Finding common genes that are co-regulated in four different species is not evidence for heritable gene expression. The authors have not actually shown that any of these traits are heritable. This is a hypothesis based on likelihood and should be presented as that.
- Some conclusion sentences contradict later results or figures legends. Wording should be adjusted accordingly. For example, "These results suggest that activated SA signalling is responsible for sustained transcriptional reprogramming in *A. thaliana*" vs. "SID2-dependent SA accumulation is not required for sustained 1279 transcriptome responses in *A. thaliana*".

Reviewer #3 (Comments for the Author):

In this paper, the authors study gene expression patterns related to early pattern triggered immunity in Brassicaceae. They used the MAMP flg22 to emulate PTI and screened early transcriptional differences in a set of several Arabidopsis accessions and several more or less closely related other Brassicaceae. The findings suggest conservation of a subset of responses/response genes, as evidenced by similar responses in all tested Brassicaceae. Interestingly, additional sets of genes seem to show diversification in the responses, as they are upregulated only in specific species. Lastly, the authors measure secondary metabolite responses. Maybe a bit unsurprisingly, they do find some species specific metabolites that are produced after flg22 treatment.

The authors explore the annotations of these genes, identify associated transcription factor binding sites and explore links with sequence evolution. Overall, I think this is an interesting study that warrants publication. The datasets are well done. Analysis is deep and the findings are interesting. Some are negative, but that is also a result. There are a couple of points that I think should be addressed prior to publication.

One major point is the obvious presence of species or accession specific data that is not picked up by this analysis, because the study relies on a read mapping approach. Seeing the complexity of the data, this could result in several problems. One is misidentification of orthologs. In the paragraph around line 311, the authors address incorrect ortholog assignment, so I think that is dealt with properly. The other one is mis mapping of genes that are actually not true orthologs to the reference. E.g. Capsella has a completely neofunctionalized gene (possibly in a cluster of two or more) that is involved in flg22 perception that does not occur in *A. thaliana.* How can the authors be sure that the reads for this gene are not mapped to the single *A. thaliana* copy and thus affecting the DEG analysis? Could the authors generally estimate how much data is lost due to the read mapping approach?

l179 could the authors elaborate whether their RBBH approach could show any bias or problems due to gene duplications or CNV events? In other words, does the set contain genes of which there definitely only is 1:1 orthology, or are there genes where there is for example one copy in Arabidopsis and 2 copies in Capsella? If so, how was that dealt with?

l246 here you write almost complete lack, whereas in the next lines you try to find some categories. However of the mentioned ones, I only see phenylpropanoid metabolic process colored. Moreover, this seems like a fishing exercise to me. Trying to highlight something for the sake of highlighting it. Wouldn't it be just fine and justifiable to state that there are no GOs enriched in the individual species gene sets? To me this would hint at much more specialized processes and would link well with the metabolite data presented later.

Also Figure 3D, if I understand the figure correctly, the split GO analysis is rather meaningless, because none of the circles show significant p-values (all are white). It might be a matter of taste, but I would say such results could be presented in a table or differently, leaving more space for the other figure panels. All the figures already require a lot of zooming in.

Some statements are rather strongly worded. This relates to explanations/introductions as well as to some of the findings. Toning down some of those would be recommended. E.g.

l113 FLS2 is conserved? Is it really, or is it under balancing selection? I don't know and no sources are cited. Safest would be to say: FLS2 homologs exist in all Brassicaceae, Solanaceae and Poaceae.
l151 there is no evidence for the ETI claim, several other quantitative effects might also lead to this phenotype. In fact, wouldn't the experiment with the hrcC mutant suggest that ETI is not involved?
l155 how can you conclude that PTI is limiting the growth. If a certain effector is essential for virulence (e.g. host target manipulation) and that effector cannot be secreted in the mutant, it is obvious that it will not grow, and this has nothing to do with whether the plant shows a defence response.
I'd remove the overinterpretation of the two last statements. The conclusion (that the results of flg22 triggers/treatments differ) is interesting enough.

l183, 868 genes are DEG between spp. Is this at the same time point or at any time point? Would this make a difference?
l213, Is the really purifying selection? could be balancing?
l410, I do not understand what you are trying to measure
l447 I don't think that with just these measures one can conclude that these genes are under balancing selection. The frequency of mutations in the populations would need to be estimated. It would be possible to do this from the 1001 genome data. Alternatively, toning down of the statements and explaining that other measures would be needed to really assess balancing selection would imo suffice here too.
l569. I don't really understand what you're trying to say in the summary paragraph.

**TPC2020-LSB-00600R1 1st Revision received Oct. 30, 2020**

Reviewer comments and **author responses**:

Reviewer #1:

Point 1. I have a number of concerns about the inference of selection made in this paper. First, the abstract argues that there is a set of genes with expression responses under purifying selection in Brassicaceae. However, the main result for this claim seems to be that the 868 genes that show differential expression in response to flg-22 in the species studied. However, without any model for the amount of conservation of expression, it's not clear to me why 868 is more than expected under neutrality. To make this claim, the authors need to provide evidence that the number of genes with conserved expression responses is more than expected under drift. Otherwise, I think the strongest statement would be that conservation of the response is "consistent with neutral evolution or stabilizing selection" (I generally hear 'purifying selection' used to describe selection against new mutations, so this means selection on specific genetic variants. I think for a trait like gene expression, it would be more accurate to say "stabilizing selection" constrains trait evolution. But I realize that these terms are used in different ways.)

**RESPONSE: We agree with the reviewer that the term "purifying selection" should be reserved for selection against allelic variants and "stabilizing selection" for phenotypic traits. We therefore have removed claims related to evolutionary selection on gene expression changes.**

Point 2. Second, the abstract claims that many genes show species-specific expression signatures. The evidence for this claim comes from an analysis comparing phylogenetic distances to a PCA of differentially expressed genes. The manuscript argues that because the PCA does not match phylogenetic differences there must have been directional

selection (Fig 5e). However, I disagree that PCA of the expression of all differentially-expressed-in-flg-22 genes is the correct method to use here since it will be dominated by the genes with the most expression variation between species, potentially contributing to a distorted view of how much evolution there has been. This conclusion would be convincing if the manuscript used phylogenetic methods to test for selection on specific branches of the phylogeny (for an example see Nourmohammad et al. 2017 https://doi.org/10.1016/j.celrep.2017.07.033 and citations within). I also think that looking gene-by-gene would be a better approach than using a PCA.

> **RESPONSE: For clarity, the conclusion that a considerable portion of the transcriptome exhibited a species-specific signature was inferred from the clustering analyses of the expression datasets and not from the PCA. We concur to the comment that our analyses are tilted towards genes with large expression variances, which may constrain our evolutionary standpoint. Hence, we have conducted hierarchical clustering of the $\log_2$ fold changes of all 1:1 orthologues, which includes both DEGs and non DEGs at 1 h after flg22 treatment. Our analyses show species specific clustering with strong support (all bootstrap supports =100), and the relationship based on expression profile among these species is discordant to the species phylogeny (Figure 5E; bootstrap supports > 90). We have replaced the previous PCA with this result.**

Point 3. The manuscript claims that there is stronger purifying selection acting on the upstream regions of genes in cluster 5 (highly induced in all species) than for other clusters (paragraph starting line 426). However, this result is not very convincing without a statistical comparison at least showing that the clusters differ from each other. I'm also curious why the authors chose *thaliana-lyrata* divergence here instead of divergence between more distantly related species, since it may be that *thaliana* and *lyrata* are too recently diverged to see large differences in constraint between genes.

> **RESPONSE: Thank you for pointing this out. We have conducted a one-way ANOVA on the cluster-specific genetic divergence values of upstream regions. The results have been added to new Supplementary Figure 9 and show that the upstream region of conserved responsive genes have significantly lower diversity than species-specific responsive genes (although the effect is less clear for cluster 2 than cluster 5). The *thaliana-lyrata* divergence: the analysis presented in new Supplementary Figure 9 aimed at comparing the evolutionary constraint on upstream regulatory regions between *A. thaliana* – specific responsive genes (cluster 1 and 12) and phylogenetically conserved genes (cluster 2 and 5). Further clusters in the figures can be considered as controls here. The *thaliana-lyrata* whole genome pairwise alignment allows such calculation to be done and ensures that most upstream regions remain similar enough to be correctly aligned. We agree that future analyses may benefit from phylogenetic analyses of upstream regions in all four species.**

Point 4. In the "Variation in coding sequences show no strong correlation with transcriptome variation" section, the authors investigate whether sequence divergence correlates with expression level. They use total sequence similarity to measure sequence conservation, but much of the literature (including cited paper Hunt et al.) are looking specifically at dN and dN/dS. Could this explain why the authors here do not see a relationship? I'd also encourage the authors to cite more of the plant literature on this topic (for example Slotte et al. 2011 https://doi.org/10.1093/gbe/evr094, Hodgins et al 2016 https://doi.org/10.1093/molbev/msw032 and others). I'd also note that the available evidence suggests that the relationship between sequence divergence and expression seems to be due to stronger negative selection on more highly selected genes, so it's also perhaps unsurprising to not see this relationship in expression that is induced in specific environments.

> **RESPONSE: We would like to thank the reviewer for raising this issue. We have now tested if changes in expression levels correlate specifically with *dN* and *dS* (new Supplementary Figure 8). As for overall amino acid sequence identity, we observed no strong correlation between flg22-induced expression changes and *dN, dS,* or *dN*/*dS*. We have now added this analysis as new Supplementary Figure 8 to which we make reference in the relevant section. In addition, we have included further references from the plant literature on this topic.**

Point 5. This manuscript treats differential expression as binary -- either a gene is differentially expressed (adjusted p < 0.01 and minimum fold change is 2) or it isn't differentially expressed. However, I wonder if this choice means that the authors are missing out on shared transcriptome responses where in one species the response is just slightly below their cutoff. One potential way around this would be to see how robust the results are to changes in the cutoff used to measure differential expression. Alternatively, the authors could investigate the correlation in responses between species for all genes, not just those meeting their significance cutoff.

**RESPONSE: The identification of flg22-responsive genes with species-specific signatures were not solely based on a binary selection. We selected all genes with an 1:1 orthologue among all species tested and extracted the data for all of these genes that were differentially expressed at least in one species. This dataset was used for clustering. This means that some of the selected genes may be marginally or not significantly affected by the treatment in some of the species. This analysis revealed a core set of genes that displayed a species-specific pattern as visualized in Figure 5. Additionally, we have included correlations of expression changes of all the 1:1 orthologous genes (DEGs and non DEGs) and were set over with the phylogenetic relationship. We have replaced the previous PCA with this analysis (Figure 5E).**

Point 6. I am curious about what is being lost by only looking at genes with orthologs across all species. I understand that it's harder to compare these, but it would be useful to at least know what percentage of differentially expressed genes are not being included because they lack orthologs.

**RESPONSE: Thank you for the suggestion. We have compared proportions and degree of expression changes of DEGs between 1:1 orthologues and non 1:1 orthologues. The result shows no obvious difference between these two groups. This result has been included in new Supplemental Figure 2.**

Point 7. The manuscript reports that genes with shared differential expression across species were enriched for relevant GO terms, but that genes with species-specific responses did not show many GO enrichments (lines 244-248). Could this just be the result of the fact that GO terms are based on function as found in Arabidopsis so, if a gene is important for defense response in a non-Arabidopsis species only, it won't be annotated? I also find it confusing that this paragraph reports GO terms for the species-specific cluster even though there is not a significant enrichment.

**RESPONSE: We have deleted sentences related to GO enrichment analysis for clusters showing species-specific expression signatures and have added a sentence "This could be because genes showing species-specific patterns are involved in a collection of biological processes or because our GO analysis were based on functional annotations in *A. thaliana*."**

Point 8. The methods section is missing key information. I couldn't find information about the sample size for the differential expression experiments.

**RESPONSE: Thank you for pointing out this shortfall. All RNA-seq experiments were independently performed three times and all the biological replicates were included in all of the analyses. We have now explicitly described this in the Materials and Methods.**

Point 9. I also couldn't find any information in the methods for the section starting on line 424. It is essential to know how they measured divergence between *A. thaliana* and *A. lyrata,* along with how Ka/Ks and pi_a/pi_s were calculated from the 1001 genome data.

**RESPONSE: We apologize for these lapses. We have now included the procedures in the Materials and Methods section and made the code publicly available.**

<u>Reviewer #2:</u>

Point 1. In the manuscript, the authors dissect the MAMP responses of four different Brassica species.
They did transcriptomic analyses to identify differentially expressed genes (DEGs) at 1h, 8h and 24h post flg22 treatment. The genes were clustered into groups of genes that were either differentially expressed in all four species or in a species-specific manner. While a large number of these commonly differentially expressed genes are known flg22-responsive genes with an established role in immunity, around 50% of these genes either do not have an established role in immunity or are unannotated. This study therefore has the potential to provide a large set of genes with a possible important role in immunity. However, since quite many of the genes that show differential expression in a species-specific manner are associated with processes distinct from plant immunity, the number of new plant immunity genes could be substantially lower than the total number of species-specific DEGs identified in this study.

**RESPONSE: We have provided an evidence that many of the genes that have not been previously implicated in immunity are actually responsive to flg22 treatment. These genes can be important regulators of plant immunity to be investigated. We believe that our dataset is an important source of information for future studies.**

Point 2. They also analyzed the gene expression changes in several geographically diverse Arabidopsis accessions. In addition to the gene expression variation, the authors also looked at genetic variation upstream of coding regions and transcription factor binding sites. However, none of these analyses yielded any interesting observations, so they could be replaced.

**RESPONSE: We think that these analyses provide valuable pieces of information. Therefore, we would like to keep them.**

Point 3. Much more interesting is the metabolome profiles of the Brassicaceae. This is a very interesting dataset and can be expanded on more. I think this would be of very great interest to the community and can replace some of the poorer evolutionary analyses.

**RESPONSE: Thank you for emphasizing the importance of metabolite profiling in these kinds of studies. The most logical step is to identify the compounds that are specifically accumulated upon flg22 treatment, and we think this is for a future study that we would like to pursue.**

Point 4. The authors suggest that the regulation of the common set of differentially expressed genes is under purifying selection. However, they later analyse the regulatory regions of these gene clusters and don't find differences in genetic variation between clusters of DEGs. This seems to indicate that the mechanisms driving differential gene expression can't be explained by evolutionary pressure. Overall, the findings don't necessarily support the conclusions drawn on evolutionary mechanisms and pressures. I would suggest the authors rewrite the manuscript and soften some of their conclusions.

**RESPONSE: We have removed claims related to evolutionary selection on gene expression changes.**

Point 5. Finding common genes that are co-regulated in four different species is not evidence for heritable gene expression. The authors have not actually shown that any of these traits are heritable. This is a hypothesis based on likelihood and should be presented as that.

**RESPONSE: We have removed the word "heritable" throughout the text.**

Point 6. Some conclusion sentences contradict later results or figures legends. Wording should be adjusted accordingly. For example "These results suggest that activated SA signalling is responsible for sustained transcriptional reprogramming in *A. thaliana*" vs. "SID2-dependent SA accumulation is not required for sustained 1279 transcriptome responses in *A. thaliana*".

**RESPONSE: Our initial findings suggested that SA signalling pathway was responsible for the sustained transcriptional reprogramming in *A. thaliana*. However, further genetic inspection revealed that the prolonged transcriptional reprogramming was similar between wild-type and SA deficient mutants, which supports the alternative conclusion that SA alone does not explain the distinct temporal dynamics of transcriptional reprogramming in these Brassicaceae species.**

Reviewer #3:

Point 1. One major point is the obvious presence of species or accession specific data that is not picked up by this analysis, because the study relies on a read mapping approach. Seeing the complexity of the data, this could result in several problems. One is misidentification of orthologs. In the paragraph around line 311, the authors address incorrect ortholog assignment, so I think that is dealt with properly. The other one is mis mapping of genes that are actually not true orthologs to the reference. E.g. Capsella has a completely neofunctionalized gene (possibly in a cluster of two or more) that is involved in flg22 perception that does not occur in A thaliana. How can the authors be sure that the reads for this gene are not mapped to the single A thaliana copy and thus affecting the DEG analysis? Could the authors generally estimate how much data is lost due to the read mapping approach?

**RESPONSE: We apologize for the confusion. We mapped RNA-seq reads to their own genomes (i.e. RNA-seq reads from *C. rubella* were mapped to *C. rubella* genome). To clarify this point, we have added a sentence in Results section "The RNA-seq reads were mapped to their own genomes.".**

Point 2. l179 could the authors elaborate whether their RBBH approach could show any bias or problems due to gene duplications or CNV events? In other words, does the set contain genes of which there definitely only is 1:1

orthology, or are there genes where there is for example one copy in Arabidopsis and 2 copies in Capsella? If so, how was that dealt with?

**RESPONSE: The analyses performed on the set of 1:1 orthologues was limited to gene pairs with a best bi-directional (reciprocal) hit. Genes with a one-directional best hit relationship (e.g. as a result of a duplication event in only one species) were not included in the 1:1 orthologue dataset. Given that paralogous genes resulting from a duplication event might conserve the same function as the orthologous gene in other species or, by contrast, acquire a new function (neo-functionalization), we reasoned that keeping only 1:1 orthologues was more adequate than alternative approaches such as averaging expression values across paralogues within a species. We have now clarified this point further in the corresponding methods section.**

Point 3. l246 here you write almost complete lack, whereas in the next lines you try to find some categories. However of the mentioned ones, I only see phenylpropanoid metabolic process colored. Moreover, this seems like a fishing exercise to me. Trying to highlight something for the sake of highlighting it. Wouldn't it be just fine and justifiable to state that there are no GOs enriched in the individual species gene sets? To me this would hint at much more specialized processes and would link well with the metabolite data presented later.

**RESPONSE: We have removed sentences related to the GO enrichment analysis for the species-specific clusters. Conversely, we then placed a statement "This could be because genes showing species-specific patterns are involved in a collection of biological processes or because our GO analysis were based on functional annotations in *A. thaliana*."**

Point 4. Also Figure 3D, if I understand the figure correctly, the split GO analysis is rather meaningless, because none of the circles show significant p-values (all are white). It might be a matter of taste, but I would say such results could be presented in a table or differently, leaving more space for the other figure panels. All the figures already require a lot of zooming in.

**RESPONSE: We would like to keep this figure as it shows that many GO terms are enriched in conserved genes but almost no GO term enrichment for species-specific responsive genes.**

Point 5. Some statements are rather strongly worded. This relates to explanations/introductions as well as to some of the findings Toning down some of those would be recommended. E.g.
l113 FLS2 is conserved? Is it really, or is it under balancing selection? I don't know and no sources are cited. Safest would be to say: FLS2 homologs exist in all Brassicaceae, Solanaceae and Poaceae.

**RESPONSE: We have modified the text as follows. "For instance, genomes of many plant lineages including families of Brassicaceae, Solanaceae and Poaceae contain *FLS2*, whereas…"**

Point 6. l151 there is no evidence for the ETI claim, several other quantitative effects might also lead to this phenotype. In fact, wouldn't the experiment with the hrcC mutant suggest that ETI is not involved?

**RESPONSE: We did not claim that *Pto* DC3000 triggers ETI in *E. salsugineum*. Our *Pto* DC3000 *hrcC* data does not support ETI triggered by *Pto* DC3000 or exclude this possibility. Our data showed that flg22 treatment does not restrict growth of *Pto* DC3000 (may elicit ETI) and *Pto* DC3000 *hrcC* (does not elicit ETI) in *E. salsugineum*, thus indicating that only ETI (even if it is triggered) does not explain the phenotype.**

Point 7. l155 how can you conclude that PTI is limiting the growth. If a certain effector is essential for virulence (e.g. host target manipulation) and that effector cannot be secreted in the mutant, it is obvious that it will not grow, and this has nothing to do with whether the plant shows a defence response.
I'd remove the overinterpretation of the two last statements. The conclusion (that the results of flg22 triggers/treatments differ) is interesting enough.

**RESPONSE: We have removed this sentence.**

Point 8. l410, I do not understand what you are trying to measure.

**RESPONSE: The relationship between gene expression and coding sequence evolution appears to be species- or condition-dependent. Therefore, we asked whether the variation in basal or flg22-triggered expression changes is correlated with variation in amino acid sequences among the tested Brassicaceae species.**

Point 9. l447 I don't think that with just these measures one can conclude that these genes are under balancing selection. The frequency of mutations in the populations would need to be estimated. It would be possible to do this from the 1001 genome data. Alternatively, toning down of the statements and explaining that other measures would be needed to really assess balancing selection would imo suffice here too.

**RESPONSE: We have toned down the statement. In addition, we used the frequencies of mutation in the 1001 genome data.**

**TPC2020-LSB-00600R1 2nd Editorial decision – *revision requested* Dec. 14, 2020**

As you will see in the comments below, from the reviewers, there are some requests for modifications to the text and the addition of some experimental details to enable future researchers to repeat the work. We ask that you attend to these requests. The revisions will be evaluated by the editor to assess if there is a need for external input or if they were sufficient to address the concerns and merit acceptance.

------------------------------------------------------------------------------ Reviewer comments:
Reviewer #1 (Comments for the Author):

The authors have responded to many of my concerns satisfactorily. However, there were a few that were not adequately addressed:

1. I am still skeptical of the analysis of selection on the transcriptome. I appreciate that the authors have clarified that their conclusions depend on a clustering analysis, not on the PCA, and that they have adjusted the figure to show the clustering analysis. However, the clustering analysis suffers from the same issue as the PCA. It is not clear how different gene expression levels contribute to the overall patterns summarized by the clustering, especially because the authors are making the clusters based on log-fold expression changes. We have an expectation that quantitative trait variation will follow phylogenetic patterns under drift, because quantitative traits are made up of the effects of multiple loci that themselves follow phylogenetic patterns of allele frequency change. However, these clusters of log-fold changes are not additive combinations of alleles, so I don't know that we can expect the clustering to follow phylogenetic patterns under neutrality. I still think it would be more robust to do a gene-by-gene test as has been done by other studies looking at selection on gene expression level.

2. I'd asked the authors to provide a statistical test backing up their claim that there is lower genetic divergence in the upstream regions of genes in cluster 5 than on other genes. The revised manuscript includes an ANOVA comparing upstream genetic divergence for genes of different clusters. However, I can't find any information about this ANOVA in the methods section, so I can't evaluate it. For example, it's unclear to me what the sample size is, or what windows have been included. Along with a thorough description in the methods, Figure S9's legend needs to describe the inset with the ANOVA as well.

3. The text states "A small number of genes with Ka/Ks and PI_a/PI_s ratios larger than 1 was observed in each cluster (Supplemental Figure 11), indicating that positive selection acts on some flg22-responsive genes in A. thaliana" (lines 433-436). However, PI_a/PI_s > 1 does not indicate positive selection, it indicates relaxed negative selection. Figure S11 shows that clusters 1, 7, and 9 do not have any genes with Ka/Ks > 1, so the statement in the manuscript is incorrect. If the authors really want to combine the polymorphism data (pi) with divergence data (Ka and Ks) to detect positive selection, they should do a MacDonald-Kreitman test.

Reviewer #3 (Comments for the Author):

In this revision the authors have tried to address a lot of comments by all reviewers. Most of my comments have been addressed.
The methods on read mapping and some other technical confusions that could make the study invalid have been clarified.

The large number of overstatements is gone. This is a good thing, but unfortunately, rather than tackling the comments by performing rigorous additional (statistical) testing, it feels like the conclusions have been toned down throughout almost all of the manuscript. After reading this version, the results and conclusions seem less exciting. There is a lot of underlying data and the expression analyses and motif searches are done very well, but the paper does not convey the kind of exciting evolutionary massage anymore that it did before.

When reading the new version, I spotted a few things that warrant attention.
The line numbers that I cite below refer to the version in which the changes are tracked.

l367 no statistical support is presented
p-values should be presented for the correlations in S Fig 8

In the new version of the main text the authors talk in one section about dN/dS (l468), in a later section (l492 onward) about Ka/Ks. I do hope the authors know that these are different names for the same statistic!

l494 I don't see how this claim is backed up by the SFS in S Fig 10.
S Fig 10 is presented without any explanation.

Related to that. Maybe I am overlooking something, but if the authors want to calculate possible selective pressure on the involved genes within A thaliana, why don't they perform a proper McDonald Kreitman Test? In S Fig 11, it looks like the authors just highlighted some genes that suit their claims.
l499 is this small number larger than expected under a neutral demographic scenario? If not, does this mean anything at all?

The method for dNdS calculation is not quite clear to me. Which sequences were included?
Similar piN/PiS.

---

**TPC2020-LSB-00600R2 2nd Revision received — Jan. 15, 2021**

---

Reviewer comments and **author responses**:

Reviewer #1:

Point 1. I am still skeptical of the analysis of selection on the transcriptome. I appreciate that the authors have clarified that their conclusions depend on a clustering analysis, not on the PCA, and that they have adjusted the figure to show the clustering analysis. However, the clustering analysis suffers from the same issue as the PCA. It is not clear how different gene expression levels contribute to the overall patterns summarized by the clustering, especially because the authors are making the clusters based on log-fold expression changes. We have an expectation that quantitative trait variation will follow phylogenetic patterns under drift, because quantitative traits are made up of the effects of multiple loci that themselves follow phylogenetic patterns of allele frequency change. However, these clusters of log-fold changes are not additive combinations of alleles, so I don't know that we can expect the clustering to follow phylogenetic patterns under neutrality. I still think it would be more robust to do a gene-by-gene test as has been done by other studies looking at selection on gene expression level.

> **RESPONSE: We agree that a gene-by-gene test for testing evolutionary selection adds value to this manuscript. Therefore, we have performed the multi-optima phylogenetic Ornstein-Uhlenbeck modelling (Hansen, 1997) in each orthologue. We have used the $\log_2$ fold changes as trait values to be fit because our major interest in this manuscript is the evolution of gene expression changes in response to flg22. In addition, because this is a gene-by-gene analysis in which each gene contributes to the overall patterns equally, results are expected to be robust against biases, for example, those potentially caused by different expression levels or by different scales of fold changes among genes. In these models, the strength of neutral drift and the pull towards the estimated theoretical optimum were taken into account with parameters $\sigma^2$ and $\alpha$, respectively. Potentially adaptive changes were**

**searched as regime shifts of the theoretical optimum by the phylogenetic LASSO algorithm with a phylogeny-aware information criterion (pBIC) (Khabbazian et al., 2016). The regime shifts were detected in 3,136 out of 5,961 orthologous genes, suggesting frequent evolutionary changes in flg22-triggered transcriptional responses that could potentially be selectively driven (new Supplementary Figure 7). Notably, the four species-specific clusters (Figure 5A) showed the highest shift frequencies compared with the others in the branches connected to the corresponding species (new Supplementary Figure 7). These results suggest that those clusters enrich a group of genes that could have evolved through the non-neutral switching of selective regimes in addition to neutral drift and/or stabilizing selection. New Supplementary Figure 7 has been added and the text has been modified accordingly**

Point 2. I'd asked the authors to provide a statistical test backing up their claim that there is lower genetic divergence in the upstream regions of genes in cluster 5 than on other genes. The revised manuscript includes an ANOVA comparing upstream genetic divergence for genes of different clusters. However, I can't find any information about this ANOVA in the methods section, so I can't evaluate it. For example, it's unclear to me what the sample size is, or what windows have been included. Along with a thorough description in the methods, Figure S9's legend needs to describe the inset with the ANOVA as well.

**RESPONSE: Thank you for the comment. We have added more information about the one-way ANOVA in the materials and methods (section intraspecific variability) and to the legend of Supplementary Figure 9 (now Supplementary Figure 10). The sample sizes in each group have also been added to this figure (in the boxplots). The R script for the ANOVA and its visualization can be found at https://gitlab.mpcdf.mpg.de/slaurent/mk_dfe.git in the folder *visualization*.**

Point 3. The text states "A small number of genes with Ka/Ks and PI_a/PI_s ratios larger than 1 was observed in each cluster (Supplemental Figure 11), indicating that positive selection acts on some flg22-responsive genes in A. thaliana" (lines 433-436). However, PI_a/PI_s > 1 does not indicate positive selection, it indicates relaxed negative selection. Figure S11 shows that clusters 1, 7, and 9 do not have any genes with Ka/Ks > 1, so the statement in the manuscript is incorrect. If the authors really want to combine the polymorphism data (pi) with divergence data (Ka and Ks) to detect positive selection, they should do a MacDonald-Kreitman test.

**RESPONSE: We agree. We have conducted a McDonald-Kreitman test for each gene in Supplementary Figure 11. The results of the MK-test showed no single significant gene after correcting for multiple testing, and we have therefore removed the figure and corresponding text. We note that patterns of expression can potentially be affected by genetic variation in functional non-coding regions while only coding variation has been considered in this MK-test.**

<u>Reviewer #3:</u>

Point 1. l367 no statistical support is presented.

**RESPONSE: We used q-value < 0.01; |log2 fold change| > 1 criteria as stated in the Figure legend.**

Point 2. In the new version of the main text the authors talk in one section about dN/dS (l468), in a later section (l492 onward) about Ka/Ks. I do hope the authors know that these are different names for the same statistic!

**RESPONSE: We agree with the reviewer. Ka/Ks was wrongly used to refer to the count number of non-synonymous and synonymous differences between two species. We note that Dn/Ds (count number in a sample of size 2) is different from dN/dS (parameter estimate from the transition matrix calibrated from a potentially large number of phylogenetic branches). When we performed McDonald-Kreitman test, we found no single significant gene after correcting for multiple testing, and we therefore have removed the figure and corresponding text.**

Point 3. l494 I don't see how this claim is backed up by the SFS in S Fig 10. S Fig 10 is presented without any explanation.

**RESPONSE: We have removed this figure.**

Point 4. Related to that. Maybe I am overlooking something, but if the authors want to calculate possible selective pressure on the involved genes within A thaliana, why don't they perform a proper McDonald Kreitman Test? In S Fig 11, it looks like the authors just highlighted some genes that suit their claims.

**RESPONSE: We agree and have conducted a MK-test for each gene in Supplementary Figure 11. No gene was significant after fd-correction for multiple testing, and we therefore have removed the figure and corresponding text. Please also see our response to point #2 from Reviewer 1 for more information.**

Point 5. The method for dNdS calculation is not quite clear to me. Which sequences were included?
Similar piN/PiS.

**RESPONSE: We used a Maximum Likelihood approach that uses a model of codon substitution described in Goldman and Yang, 1994. This method is implemented in the program *codeml*, which is part of the toolkit PAML (Yang and Nielsen, 2000). In each panel, sequences with a 1:1 orthologous relationship as inferred using the best bi-directional hit for each pair of species were included. This is described in the Method section. The Dn/Ds and pi_n/pi_s were calculated using R scripts generated for this project and publicly available at the following repository: https://gitlab.mpcdf.mpg.de/slaurent/mk_dfe.git. As no gene was significant after fd-correction for multiple testing, we therefore have removed the figure and the corresponding text.**

| TPC2020-LSB-00600R2 3rd Editorial decision – *accept with minor revision* | Feb. 8, 2021 |
|---|---|

There are some concerns about the OU work but after discussion, we agreed that simply mentioning at the start of that section that the necessarily limited species sampling might inflate the Type I error. This would give the reader the necessary information and also help to inform the reader on the scale of experiments if they are interested in something similar in the future.

The rest of the comments are asking for clarification or simply more information on certain points.

Additionally, one of the reviewers found this citation on the difficulty of PCs and phylogenetic inference that might be useful in the future: https://academic.oup.com/sysbio/article/64/4/677/1649888. I am having my lab read it and thought your group would appreciate it. This is just for future reference as MPMI goes further down a phylogenetic track and is not anything to do with the acceptance of this manuscript.

------------------------------------------------------------------------ Reviewer comments:

Reviewer #1 (Comments for the Author):

The authors have responded to my comments from the previous review. I have two follow up comments.

1) I appreciate that the authors have conducted a gene-by-gene analysis and the results are encouraging. I have a few comments about this new section

I found this statement in the review response and a similar one in the manuscript confusing "Because this is a gene-by-gene analysis in which each gene contributes to the overall patterns equally, results are expected to be robust against biases, for example, those potentially caused by different expression levels or by different scales of fold changes among genes." because it's not clear to me what the authors mean by biases that could be caused by different expression levels or scales of fold changes. Can they clarify this statement?

I am also concerned that the OU model chosen by the authors is not appropriate for the number of species here. The paper about their chosen method (Khabbazian et al.) uses many more species in its examples and Cooper et al 2016 (https://doi.org/10.1111/bij.12701) suggest that even phyologenies with 25 species can be too small to avoid type 1 errors with OU methods. It would be helpful if the authors could add some context about why they chose this particular method and why they expect it to be robust in this situation.

2) I also appreciate that the authors have added a description of the ANOVA done to compare sequence divergence between clusters to the methods. My apologies if I'm missing something but I can't find any numbers in the boxplot inset in figure s10 specifying sample size for the different clusters (as is indicated in the legend). It would be useful to clarify in the results (around line 438) that they only have identified statistically significant differences in divergence

upstream of clusters when they look at the first 100bp upstream of the gene and not at other distances. I have a few additional comments about confusing wording in the paper:

- The modified sentence "The observed divergent gene expression between different species together with the low variation within species could have been shaped by lineage-specific non-neutral evolution, including, but not limited to, stabilizing selection and adaptive evolution in addition to neutral evolution." (line 324) is very confusing. It's not clear to me what the authors intend to say here.
- I think that 'evolutional' should be replaced with 'evolutionary' on line 63.
- On line 66, I think the authors should clarify because they note previously that expression data is noisy and due to genetic and environmental effects, and then state that you need to do experiments in the same environmental conditions to detect 'gene expression variation'. Do they mean genetic gene expression variation here?
- The heading on line 425 uses the phrase "neutral ascendancy" and I don't understand what is meant here.

Reviewer #3 (Comments for the Author):

My comments have been addressed. I think this paper is fit for publication.

**TPC2020-LSB-00600R3 3rd Revision received Feb. 10, 2021**

Reviewer comments and **author responses**:

Reviewer #1:

Point 1. I appreciate that the authors have conducted a gene-by-gene analysis and the results are encouraging. I have a few comments about this new section: I found this statement in the review response and a similar one in the manuscript confusing "Because this is a gene-by-gene analysis in which each gene contributes to the overall patterns equally, results are expected to be robust against biases, for example, those potentially caused by different expression levels or by different scales of fold changes among genes." because it's not clear to me what the authors mean by biases that could be caused by different expression levels or scales of fold changes. Can they clarify this statement?

> **RESPONSE: We have modified the text (Line 336-342 in the word file with track changes). "The hierarchical clustering result could be largely affected by genes with large expression variation between species, potentially contributing a distorted view of the gene expression evolution. However, because the multi-optima phylogenetic Ornstein-Uhlenbeck modelling is a gene-by-gene analysis in which each gene contributes to the overall patterns equally, results are expected to be robust against biases from a small number of genes with large effect sizes."**

Point 2. I am also concerned that the OU model chosen by the authors is not appropriate for the number of species here. The paper about their chosen method (Khabbazian et al.) uses many more species in its examples and Cooper et al 2016 (https://doi.org/10.1111/bij.12701) suggest that even phyologenies with 25 species can be too small to avoid type 1 errors with OU methods. It would be helpful if the authors could add some context about why they chose this particular method and why they expect it to be robust in this situation.

> **RESPONSE: We have added the underlined sentence here (Line 346-347 in the word file with track changes)."Potentially adaptive changes were searched as regime shifts of the theoretical optimum by the phylogenetic LASSO algorithm with a phylogeny-aware information criterion (pBIC) (Khabbazian et al., 2016). We note that the necessarily limited number of species sampling in this study might inflate false positives. The regime shifts were detected in 3,136 out of 5,961 orthologous genes, suggesting frequent evolutionary changes in flg22-triggered transcriptional responses that could potentially be selectively driven (Supplementary Figure 7)."**

Point 3. I also appreciate that the authors have added a description of the ANOVA done to compare sequence divergence between clusters to the methods. My apologies if I'm missing something but I can't find any numbers in the boxplot inset in figure s10 specifying sample size for the different clusters (as is indicated in the legend). It would

be useful to clarify in the results (around line 438) that they only have identified statistically significant differences in divergence upstream of clusters when they look at the first 100bp upstream of the gene and not at other distances.

**RESPONSE: We apologize the error. We put a previous version of figure by mistake. We have now added the correct one with the sample size. We have also added the clarification about upstream regions used in our analysis (Line 443 in the word file with track changes). "However, cluster 5 (highly induced in all species) showed the lowest genetic divergence on its upstream regions (the first 100 bp upstream of the gene) while the neutral synonymous variation for the same cluster was the highest (Supplemental Figure 10)."**

**TPC2020-LSB-00600R3 4th Editorial decision – *acceptance pending* Feb. 10, 2021**

We are pleased to inform you that your paper entitled "Gene expression evolution in pattern-triggered immunity within Arabidopsis thaliana and across Brassicaceae species" has been accepted for publication in The Plant Cell, pending a final minor editorial review by journal staff. At this stage, your manuscript will be evaluated by a Science Editor with respect to its presentation of scientific content, compliance with journal policies, and presentation for a broad readership.

**Final acceptance from Science Editor Feb. 24, 2021**
